# Integrated metabolomics, network pharmacology and biological verification to reveal the mechanisms of *Nauclea officinalis* treatment of LPS-induced acute lung injury

**DOI:** 10.1186/s13020-022-00685-6

**Published:** 2022-11-24

**Authors:** Han Xu, Sicong Xu, Liyan Li, Yuhuang Wu, Shiying Mai, Yiqiang Xie, Yinfeng Tan, Ailing Li, Fengming Xue, Xiaoning He, Yonghui Li

**Affiliations:** 1 Key Laboratory of Tropical Translational Medicine of Ministry of Education, Hainan Provincial Key Lab of R&D on Tropic Herbs, College of Pharmacy, Hainan Medical University, No. 3 Xueyuan Road, Hainan 571199 Haikou, People’s Republic of China; 2 The Second Affiliated Hospital of Hainan Medical University, 368 Yehai Av., Haikou, 571199 Hainan People’s Republic of China; 3 College of Biomedical Information and Engineering, Key Laboratory of Tropical Translational Medicine of Ministry of Education, Hainan Medical University, No. 3 Xueyuan Road, Haikou, 571199 Hainan People’s Republic of China; 4 College of Chinese Medicine, Hainan Medical University, No. 3 Xueyuan Road, Haikou, 571199 Hainan People’s Republic of China

**Keywords:** Acute lung injury, *Nauclea officinalis*, Metabolomics, Network pharmacology, Mechanisms

## Abstract

**Background:**

Acute lung injury (ALI) is a severe inflammatory disease, underscoring the urgent need for novel treatments. *Nauclea officinalis* Pierre *ex* Pitard (Danmu in Chinese, DM) is effective in treating inflammatory respiratory diseases. However, there is still no evidence of its protective effect against ALI.

**Methods:**

Metabolomics was applied to identify the potential biomarkers and pathways in ALI treated with DM. Further, network pharmacology was introduced to predict the key targets of DM against ALI. Then, the potential pathways and key targets were further verified by immunohistochemistry and western blot assays.

**Results:**

DM significantly improved lung histopathological characteristics and inflammatory response in LPS-induced ALI. Metabolomics analysis showed that 16 and 19 differential metabolites were identified in plasma and lung tissue, respectively, and most of these metabolites tended to recover after DM treatment. Network pharmacology analysis revealed that the PI3K/Akt pathway may be the main signaling pathway of DM against ALI. The integrated analysis of metabolomics and network pharmacology identified 10 key genes. These genes are closely related to inflammatory response and cell apoptosis of lipopolysaccharide (LPS)-induced ALI in mice. Furthermore, immunohistochemistry and western blot verified that DM could regulate inflammatory response and cell apoptosis by affecting the PI3K/Akt pathway, and expression changes in Bax and Bcl-2 were also triggered.

**Conclusion:**

This study first integrated metabolomics, network pharmacology and biological verification to investigate the potential mechanism of DM in treating ALI, which is related to the regulation of inflammatory response and cell apoptosis. And the integrated analysis can provide new strategies and ideas for the study of traditional Chinese medicines in the treatment of ALI.

**Graphical Abstract:**

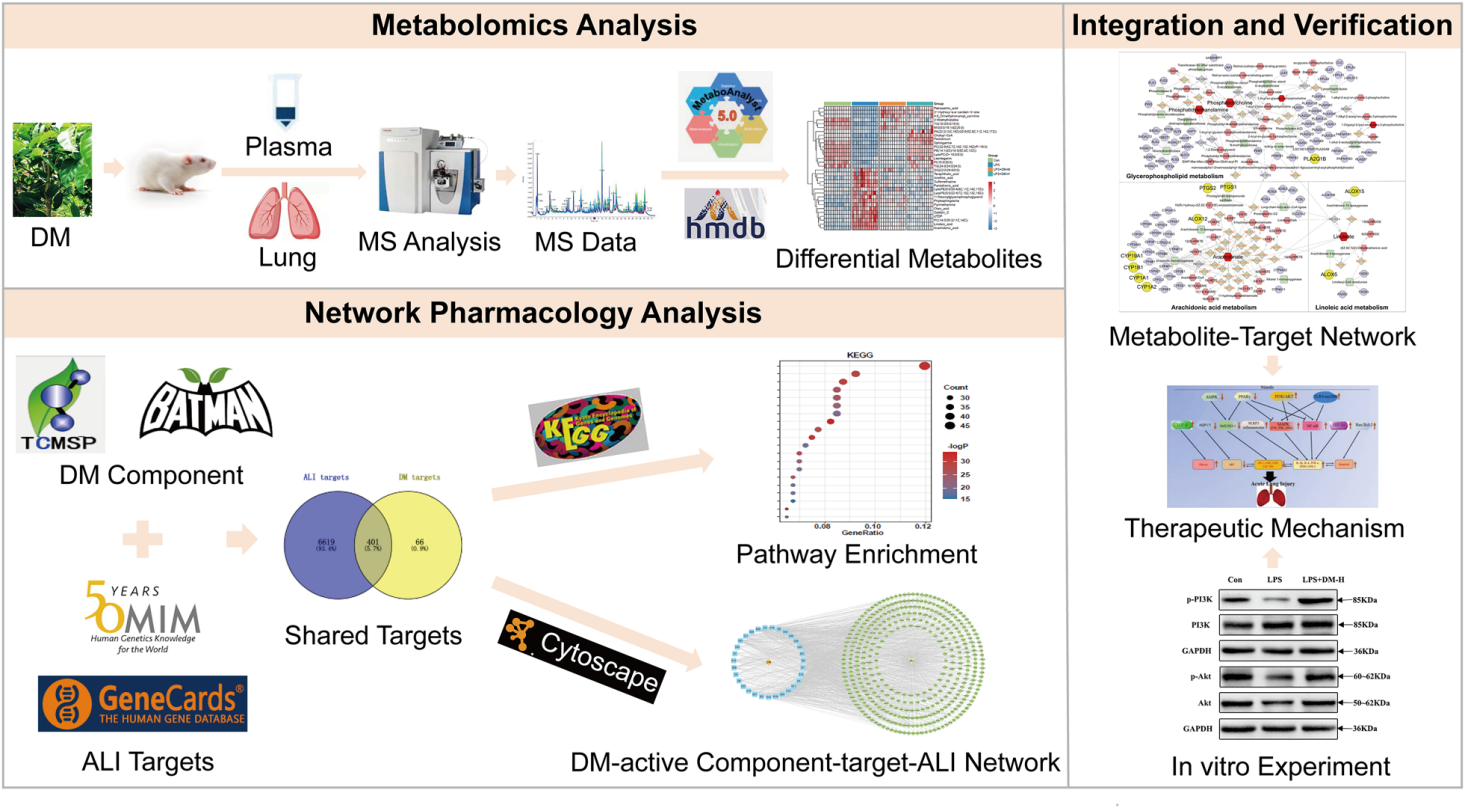

**Supplementary Information:**

The online version contains supplementary material available at 10.1186/s13020-022-00685-6.

## Background

Acute lung injury (ALI) is a critical disease with a complex pathology that is mainly characterized by inflammatory dysregulation, disruption of pulmonary endothelial and epithelial barriers, and gas exchange disorders [1, 2]. At present, the common treatments for ALI are reducing pulmonary inflammatory damage and relieving respiratory failure, and conventional treatment drugs include steroids, statins, and glucocorticoids. However, these drugs can only relieve clinical symptoms and are unable to reduce mortality from ALI [3, 4]. The mortality rate of ALI is 40% and above [5]. Therefore, it is urgent to discover effective therapeutic strategies for the treatment of ALI.

Numerous Chinese medicines have shown favorable therapeutic effects on ALI in clinical practice [6, 7]. *Nauclea officinalis* Pierre *ex* Pitard (Danmu in Chinese, DM) is a Li-folk Chinese medicine that has been used to treat inflammatory and infectious diseases for over 40 years in Hainan Province [8]. A variety of pharmacological activity studies have been conducted to determine the internal mechanism of DM. The results show that DM has comprehensive biological activities such as anti-inflammatory, antibacterial, antiviral, antioxidant, immune regulation, and promotion of cell proliferation [9]. Our previous study also demonstrated that DM extract could inhibit the release of inflammatory cytokines in RAW264.7 cells and reduce the expression of NO, TNF-α, IL-1β and IL-6 [10]. Further studies have shown that DM may repair the endothelial cell barrier of lung injury by inhibiting the inflammatory response, improving intercellular connections, and promoting angiogenesis [11]. These studies partly explain the excellent effects of DM on inflammatory diseases. However, the protective effects of DM on ALI and its underlying mechanisms remain unclear. Therefore, the study of the mechanisms of DM on ALI may be helpful in providing more options for ALI treatment.

Given the complexity of ALI pathogenesis, it is difficult to reveal the mechanisms of DM on ALI using traditional pharmacological methods. By monitoring the overall dynamic changes in metabolites in response to drug treatment, metabolomics can comprehensively reflect the development of disease and is a powerful tool for the diagnosis and treatment of complex diseases [12, 13]. Therefore, metabolomics is very suitable for revealing the mechanism of Chinese medicine in the treatment of ALI [14, 15]. However, metabolomics only reflect the changes in endogenous small molecule metabolites. The mechanism of metabolite changes remains unclear [16]. Therefore, metabolomics alone has difficulty revealing the complete therapeutic mechanism of DM on ALI.

In recent years, network pharmacology has developed rapidly in the speculation of the mechanisms of Chinese medicine [17]. Network pharmacology is frequently used to predict the targets of Chinese medicine, identify biomarkers of disease, and then speculate on the regulatory mechanisms of Chinese medicine [18, 19]. In previous literature, network pharmacology was employed to explore the therapeutic effect and potential mechanism of ginseng on ALI. The results revealed that ginseng could inhibit the PI3K/Akt and MAPK signaling pathways by downregulating the expression of STAT3, VEGFA, FGF2, PIK3CA, MAPK1 and IL2, which provides a good example of network pharmacology applied to ALI [20]. However, network pharmacology relies on public databases, and database updates seriously affect the reliability of network pharmacology results. Moreover, network pharmacology could predict potential targets and related pathways, but it is still unclear whether the predicted results are accurate. Therefore, the results also need to be verified with other methods [21].

Therefore, in this study, we integrated metabolomics, network pharmacology, and biological experimental methods of verification to reveal the influences of DM on ALI and the underlying mechanisms. First, untargeted metabolomics was applied to determine the effects of DM on ALI, and candidate metabolites and related metabolic pathways were screened. Subsequently, network pharmacology was used to predict the targets of DM active components in ALI and the pathways involved, and the regulatory metabolite-related proteins and responses were further integrated and analyzed to predict the primary mechanism of DM treatment in ALI. Finally, biological experiments were used to verify the accuracy of the predictions. In brief, this comprehensive strategy compensates for the individual defects of network pharmacology and metabolomics. This study helps us better understand the therapeutic principles of DM in the prevention and treatment of ALI.

## Materials and methods

### Reagents

LPS was obtained from Solarbio (China). Methanol, acetonitrile, and formic acid (chromatography pure) were obtained from Merck (Germany). PI3K mouse monoclonal antibody, p-PI3K rabbit monoclonal antibody, Akt rabbit polyclonal antibody, p-Akt mouse monoclonal antibody, Bcl-2 mouse monoclonal antibody, Bax mouse monoclonal antibody, GAPDH rabbit monoclonal antibody, HRP-conjugated AffiniPure goat anti-mouse IgG (H + L) and goat anti-rabbit IgG (H + L) were obtained from Proteintech Biotechnology (China).

### Source and preparation of DM

*Nauclea officinalis* Pierre *ex* Pitard stems were collected from Wuzhishan City, Hainan Province, in September 2019 and were identified by Dr. Yong-hui Li, Hainan Medical University. The voucher specimen (No. DM-20190920) was deposited at Hainan Provincial Key Lab of Research and Development on Tropic Herbs. The 5 kg DM stems were ground and extracted with water (1/10, w/v) for 2 h each time. The solutions were combined and concentrated to obtain 159 g of DM extract. The extracts were stored at 4 °C for further study.

### Animals and the lipopolysaccharide (LPS) model

Male C57BL/6J mice weighing 22–25 g were purchased from Changsha Tianqin Biotechnology Co., Ltd (Certificate number: SCXK Xiang 2022–0015). The mice were fed a standard diet and provided with water ad libitum. The animal experiment project was conducted in strict accordance with the guidelines for the care and use of laboratory animals and approved by the Ethics Committee of Hainan Medical University (No. HYLL-2022-019).

After acclimatization feeding, 90 mice were randomly divided into 5 groups (n = 18 per group): control group, LPS group, low-dose treatment group (LPS + DM-L, 100 mg/kg), medium-dose treatment group (LPS + DM-M, 200 mg/kg), and high-dose treatment group (LPS + DM-H, 400 mg/kg). The ALI model was induced by intratracheal instillation of 50 μl of LPS (5 mg/kg). The control group received normal saline. The LPS group received LPS stimulation, and the LPS + DM groups received LPS stimulation and DM treatment. After treatment for 6 h, six mice in each group were anesthetized using 3% isoflurane, and bronchoalveolar lavage fluid (BALF) was collected. After treatment for 24 h, twelve mice in each group were anesthetized, and plasma and lung tissues were collected and stored at − 80 °C for further study.

### Lung histopathology

Mouse lung tissues were immersed in 4% paraformaldehyde. The lung tissues were then dehydrated, embedded in paraffin, sliced into 4 μm sections, and stained with hematoxylin–eosin (H and E). Finally, the sections were imaged under a light microscope.

### Cytokines analysis

The levels of tumor necrosis factor-α (TNF-α), interleukin-6 (IL-6) and interleukin-1β (IL-1β) in BALF were detected using ELISA kits from Jiancheng Bioengineering Institute (Nanjing, China), according to the manufacturer's instructions.

### Plasma and lung tissue sample preparation

Blood samples were collected in heparin-coated Eppendorf tubes, held for 2 h and then centrifuged at 4 °C and 3000 rpm for 10 min to retain the supernatants as plasma samples. Lung tissue samples were homogenized in a tenfold volume of saline.

One hundred microliters of serum or 1 ml of lung tissue homogenate were diluted with a threefold volume of methanol: acetonitrile (1:1). The mixture was vortexed for 5 min, centrifuged at 4 °C and 12,000 rpm for 15 min to obtain the supernatant, and then dried at 30 °C with N_2_. Finally, the samples were reconstituted with 120 μl of 50% acetonitrile, vortexed for 5 min and centrifuged (12,000 rpm at 4 °C for 10 min) to obtain the supernatant for injection. A quality control (QC) sample was prepared from 10 μL of each test sample.

### UPLC-Q-TOF/MS analysis

The metabolomic analysis of ALI mouse plasma and lung tissue was performed using a UHPLC system (Agilent 1290, USA) coupled with a high-resolution mass spectrometer (AB Sciex Triple TOF 4600) in positive and negative ion modes. The chromatographic columns were Phenomenex Kinetex 1.7 μm XB-C18 (100A, 2.1 mm × 50 mm) and Waters Acquity UPLC BEH C18 (1.7 μm, 2.1 mm × 100 mm).

For chromatography, the mobile phase was composed of acetonitrile (A) and 0.1% formic acid (B). The gradient elution conditions for plasma were as follows: 0–1 min, 5–30% A; 1–3 min, 30–40% A; 3–5 min, 40–60% A; 5–11 min, 60–70% A; 11–13 min, 70–95% A; 13–16 min, 95% A; 16–16.1 min, 95–5% A; 16.1–20 min, 5% A. For lung tissue, the conditions were 0–1 min, 5–15% A; 1–9 min, 15–40% A; 9–10 min, 40–50% A; 10–24 min, 50–75% A; 24–25 min, 75–95% A; 25–27 min, 95% A; 27–27.1 min, 95–5% A; 27.1–32 min, 5% A. The column temperature was set at 40 °C, the flow rate was 0.3 ml/min, and the injection volume was 3 μl.

The MS parameters were as follows: the positive and negative ion modes were detected by an ESI ion source, the mass range was m/z 100–1000 Da, the ion spray voltage was + 5500/− 4500 V, and the source temperature was 550 °C. The declustering potential (DP) was ± 80 V, and the collision energy was ± 35 eV; ion source gas 1 and ion source gas 2 were set at 50 psi, and the curtain gas was set at 30 psi.

### Data processing and analysis

The raw MS data were preprocessed with Markerview 1.3.1 (AB Sciex), including peak identification, peak extraction, and peak area normalization [22]. The preprocessed data were then imported into SIMCA-P14.0 (Umetrics, Umea, Sweden) for multivariate statistical analysis and based on two parameters, R^2^Y and Q^2^, to evaluate the model truth and predictive ability. Variables were identified as differential metabolites when they satisfied the criteria VIP > 1, *P* < 0.05, FC > 1.2, or FC < 0.8 [23]. Subsequently, the secondary mass spectra of the potential metabolites were screened with Peakview 1.2.1 software and then identified by HMDB (http://www.hmdb.ca/) and KEGG (http://www.kegg.ca/). Finally, the differential metabolites were imported into MetaboAnalyst 5.0 (http://www.metaboanalyst.ca/) for metabolic pathway enrichment analysis [14].

### Network pharmacology analysis

First, all the chemical compounds of DM were obtained from the TCMSP (http://tcmspw.com/tcmsp.php) and literature review [15]. The compounds with OB ≥ 25% were selected as the active compounds of DM for further analysis. Next, the component targets were collected from BATMAN-TCM (http://bionet.ncpsb.org/batman-tcm/) [24], DrugBank (https://www.drugbank.ca/) and Swiss Target Prediction (http://www.swisstargetprediction.ch/) databases [25]. The SMILES of each compound was searched in PubChem (https://pubchem.ncbi.nlm.nih.gov/) and imported into Swiss Target Prediction (http://www.swisstargetprediction.ch/) to acquire the related targets. Moreover, DrugBank (https://www.drugbank.ca/), Gene Cards (https://www.genecards.org/), OMIM (https://omim.org/) and TTD (http://db.idrblab.net/ttd/) [26] were used to search for genes associated with ALI.

Second, the shared targets of DM active ingredients and ALI were selected, which were considered potential therapeutic targets of DM against ALI. The shared targets were imported into the STRING (https://cn.string-db.org/) database to construct a protein–protein interaction (PPI) network, and the hub genes were screened.

Finally, The Cytoscape 3.9.1 was then used to construct the “DM-active component-target-ALI” network. The target genes were then all subjected to the Database for Annotation, Visualization, and Integrated Discovery (DAVID, https://david.ncifcrf.gov/) to carry out GO and KEGG enrichment for functional enrichment analysis. *P*-values were derived from the DAVID database and set to below 0.05 [27].

### Comprehensive analysis of metabolomics and network pharmacology

The differential metabolites identified in metabolomics were imported into MetScape 3.1.3 to construct a “Metabolite-Reaction-Enzyme-Gene” network [28]. The related genes of metabolites were then combined with the target genes and metabolic pathways screened by network pharmacology to identify key metabolic pathways and core targets.

### Immunohistochemistry assay

The lung tissue sections were dewaxed and then soaked sequentially in xylene for 15 min, followed by 5 min each in different concentration of EtOH (95%, 80%, 70%, and 50%). The tissue sections were then subjected to antigen retrieval for 24 min and incubated by adding the corresponding primary and secondary antibodies. Finally, the washed sections were observed under a microscope after adding DAB chromogenic solution.

### Western blot assay

Fifty milligrams of lung tissue were homogenized with RIPA lysis buffer (containing phosphatase inhibitor). The protein content of lung tissue was quantified, and then the separation gel and concentrated gel were prepared based on the molecular weight, and a suitable sample was taken for loading. Subsequently, electrophoresis and membrane transfer were performed. The PVDF membrane was removed and blocked with 5% skim milk for 2 h, combined with the primary antibody overnight at 4 °C, and incubated with the secondary antibody for 2 h. The membrane was washed 3 times with TBST for 5 min each time and the immunoreactivity bands were detected by ChemiDoc XRS + chemiluminescence imaging system (Bio-Rad, USA).

### Statistical analysis

All data were analyzed by GraphPad Prism and presented as Mean ± SD. The different groups were evaluated by one-way ANOVA tests followed by Tukey’s multiple comparisons. *P* < 0.05 was accepted as statistically significant.

## Results

### Effects of DM on histological changes in ALI mice

**Fig. 1:**
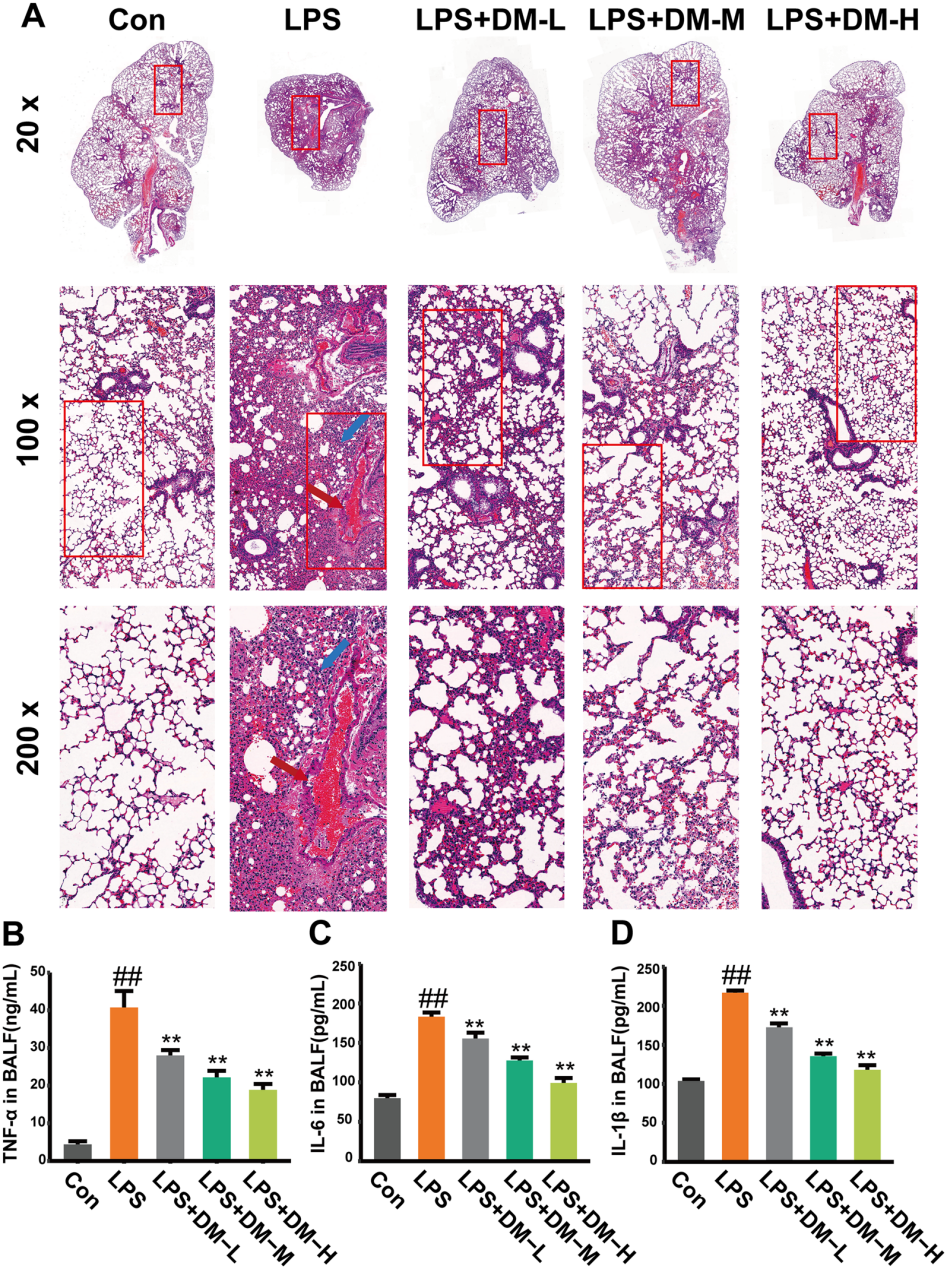
Effect of DM on histopathology and cytokines. **A** H&E staining of lung tissue in mice. Original magnification, × 20, × 100, × 200.The capillaries in the alveolar walls were congested (red arrow), and massive inflammatory cells infiltrated (blue arrow). **B** The levels of TNF-ɑ in BALF. **C** The levels of IL-6 in BALF. **D** The levels of IL-1β in BALF

To observe the effect of DM on the structural integrity of lung tissue in ALI mice, hematoxylin–eosin (H&E) staining was applied to evaluate the histological characteristics of mouse lungs. As shown in Fig. 1A, the alveolar walls of mice in the LPS group were extensively thickened, the alveolar sizes varied, the capillaries in the alveolar walls were congested (red arrow), and there was massive infiltration of inflammatory cells (blue arrow). However, lung injury was significantly improved after DM treatment. It was revealed that DM has a therapeutic effect on LPS-induced lung injury.

### DM inhibits inflammatory response in LPS-induced ALI

Pro-inflammatory TNF-α, IL-6, and IL-1β can expeditiously involve in the early phase of inflammatory response and they play a key role in the progression and pathogenesis of ALI. Therefore, their levels were quantified in BALF by ELISA (Fig. 1B–D). The levels of TNF-ɑ, IL-6, and IL-1β in the DM groups were dramatically decreased after drug treatment compared with those in the LPS model group (*P* < 0.01). The results showed that DM could improve the inflammatory response in LPS-induced ALI.

### Plasma and lung tissue metabolic profiling

When testing samples, a QC sample was inserted for every eight test samples in the injection batches to check the stability and repeatability of the instrument and method. Additional file 2: Fig. S3A–D shows the total ion chromatograms (TICs) of plasma and lung tissue in positive and negative ion modes. The overlap between each QC sample indicated that the system was stable and the operating error was small. After PCA of the QC samples, all QC samples in plasma and lung tissue showed obvious clusters (Additional file 2: Fig. S3E, F). As shown in Additional file 2: Fig. S3G, H, 88.92% and 80.14% of metabolites had an RSD% < 30% in plasma and lung tissue, respectively. These results revealed that the detection system was stable and the data were reliable.

**Fig. 2:**
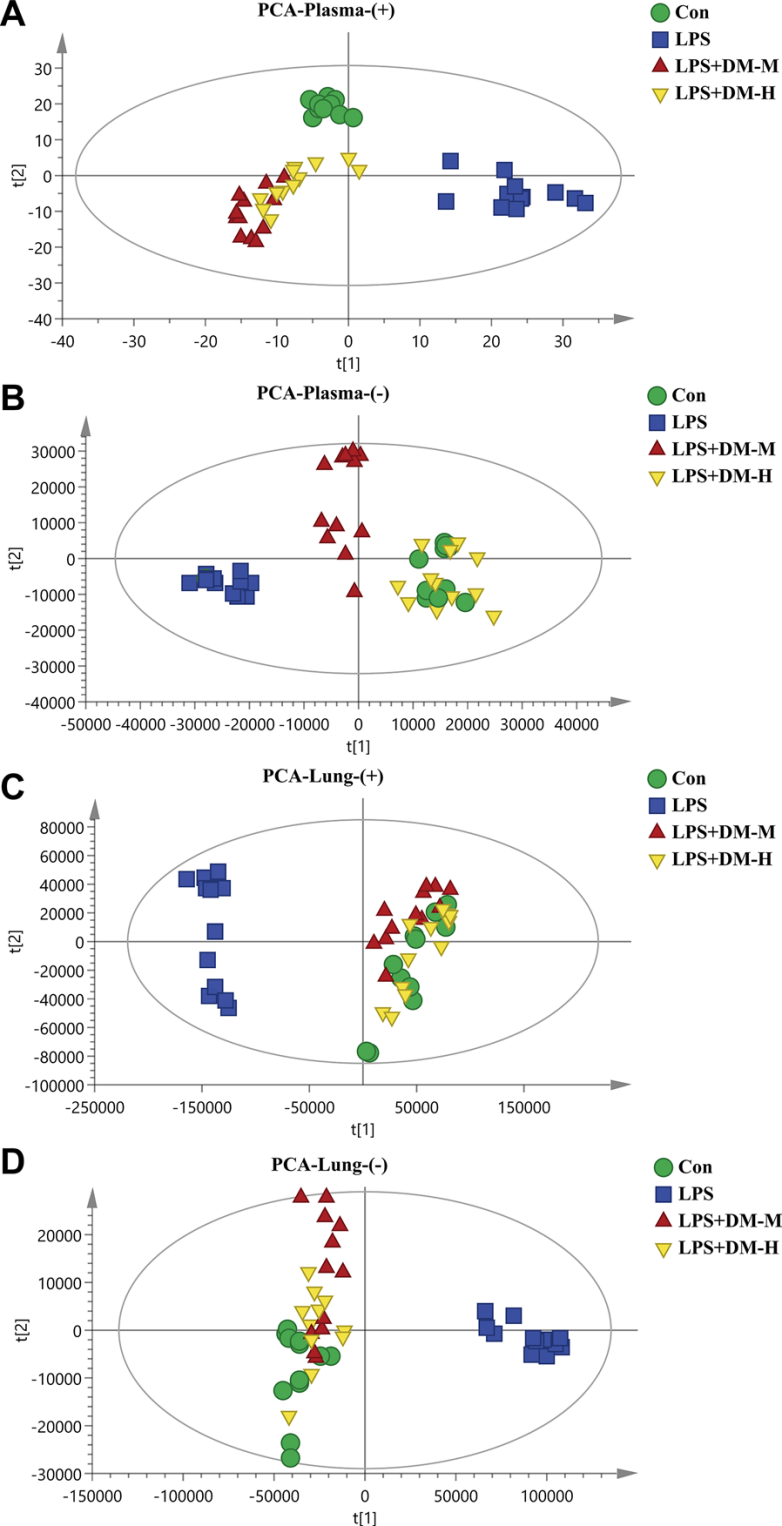
PCA analysis of plasma and lung tissue in mice. **A** Plasma positive ion mode PCA diagram; **B** plasma negative ion mode PCA diagram; **C** lung tissue positive ion mode PCA diagram; **D** lung tissue negative ion mode PCA diagram

Additional file 2: Fig. S4 shows the TICs of plasma and lung tissue samples in positive and negative ion modes. To investigate the separation among the control, LPS and DM groups, we performed PCA (Fig. 2A–D) and PLS-DA (Additional file 2: Fig. S5A–D). The samples of different groups of plasma and lung tissue of mice were well separated under the two modes, and the R^2^ and Q^2^ of the PLS-DA analysis were greater than 0.5 (Additional file 2: Table S1), which further indicated that DM had an excellent regulatory effect on the metabolism of ALI mice.

**Fig. 3:**
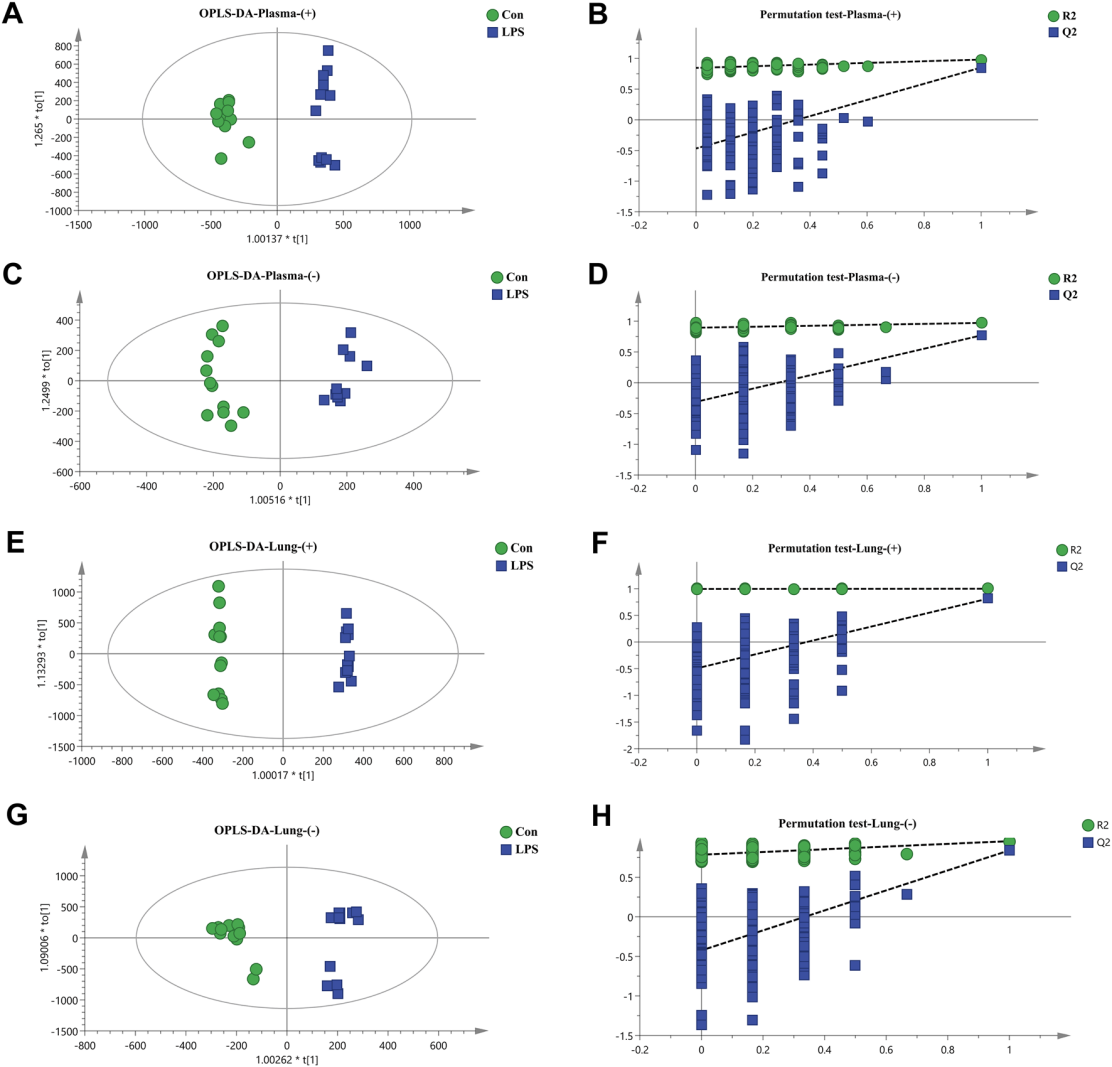
OPLS-DA and permutation test analysis of mouse plasma and lung tissue. **A** Plasma positive ion mode OPLS-DA diagram; **B** plasma positive ion mode permutation test diagram; **C** plasma negative ion mode OPLS-DA diagram; **D** plasma negative ion mode permutation test diagram; **E** positive lung tissue ion mode OPLS-DA diagram; **F** lung tissue positive ion mode permutation test diagram; **G** lung tissue negative ion mode OPLS-DA diagram; **H** lung tissue negative ion mode permutation test diagram

To further investigate the metabolic differences in ALI mice, the plasma and lung tissues from mice in the Con and LPS groups were analyzed by OPLS-DA. As shown in Fig. 3A–H, the samples of the Con and LPS groups were significantly separated in the positive and negative ion modes, indicating that the metabolism of the plasma and lung tissue of ALI mice was abnormal, the R^2^Y in the model was close to 1, and the Q^2^ was greater than 0.5, indicating that the model predictions were accurate and that the data were faithful (Additional file 2: Table S1). The permutation test showed that the models were no overfitting and reliable. Furthermore, the red dots in the S-plot away from the center might represent potential differential metabolites (Additional file 2: Fig. S6A–D).

### Identification of potential biomarkers

**Fig. 4:**
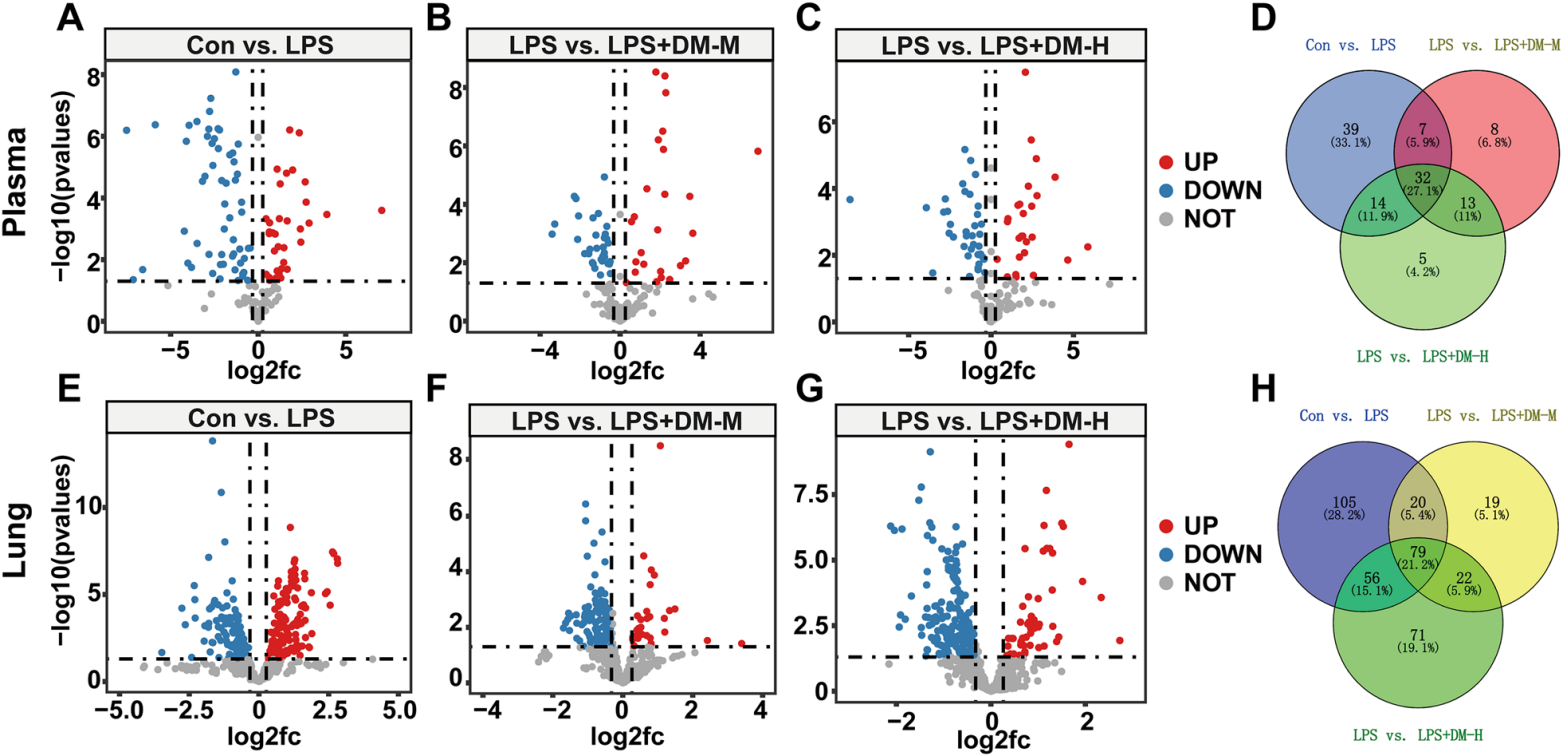
Volcano gram analysis of differential metabolites in plasma, **A** Con vs. LPS; **B** LPS vs. LPS + DM-M; **C** LPS vs. LPS + DM-H; **D** Venn diagram of differential metabolites in plasma samples. Volcano gram analysis of differential metabolites lung tissue, **E** Con vs. LPS; **F** LPS vs. LPS + DM-M; **G** LPS vs. LPS + DM-H; **H** Venn diagram of differential metabolites in lung tissue samples

Based on a VIP > 1, *p* < 0.05, FC > 1.2 or FC < 0.8, in mouse plasma samples, 36 metabolites were upregulated and 59 were downregulated in the LPS group compared with the Con group (Fig. 4A). Compared with the LPS group, there were 25 upregulated and 36 downregulated metabolites in the LPS + DM-M group (Fig. 4B) and 27 upregulated and 39 downregulated metabolites in the LPS + DM-H group (Fig. 4C). The Venn diagram showed that there were 32 differential metabolites in the 4 groups of samples (Fig. 4D). In mouse lung tissue samples, 178 metabolites were upregulated, and 150 were downregulated in the LPS group compared with the control group (Fig. 4E). Compared with the LPS group, there were 37 upregulated and 140 downregulated metabolites in the LPS + DM-M group (Fig. 4F) and 68 upregulated and 217 downregulated metabolites in the LPS + DM-H group (Fig. 4G). The Venn diagram showed 79 differential metabolites in the 4 groups of samples (Fig. 4H).

**Fig. 5:**
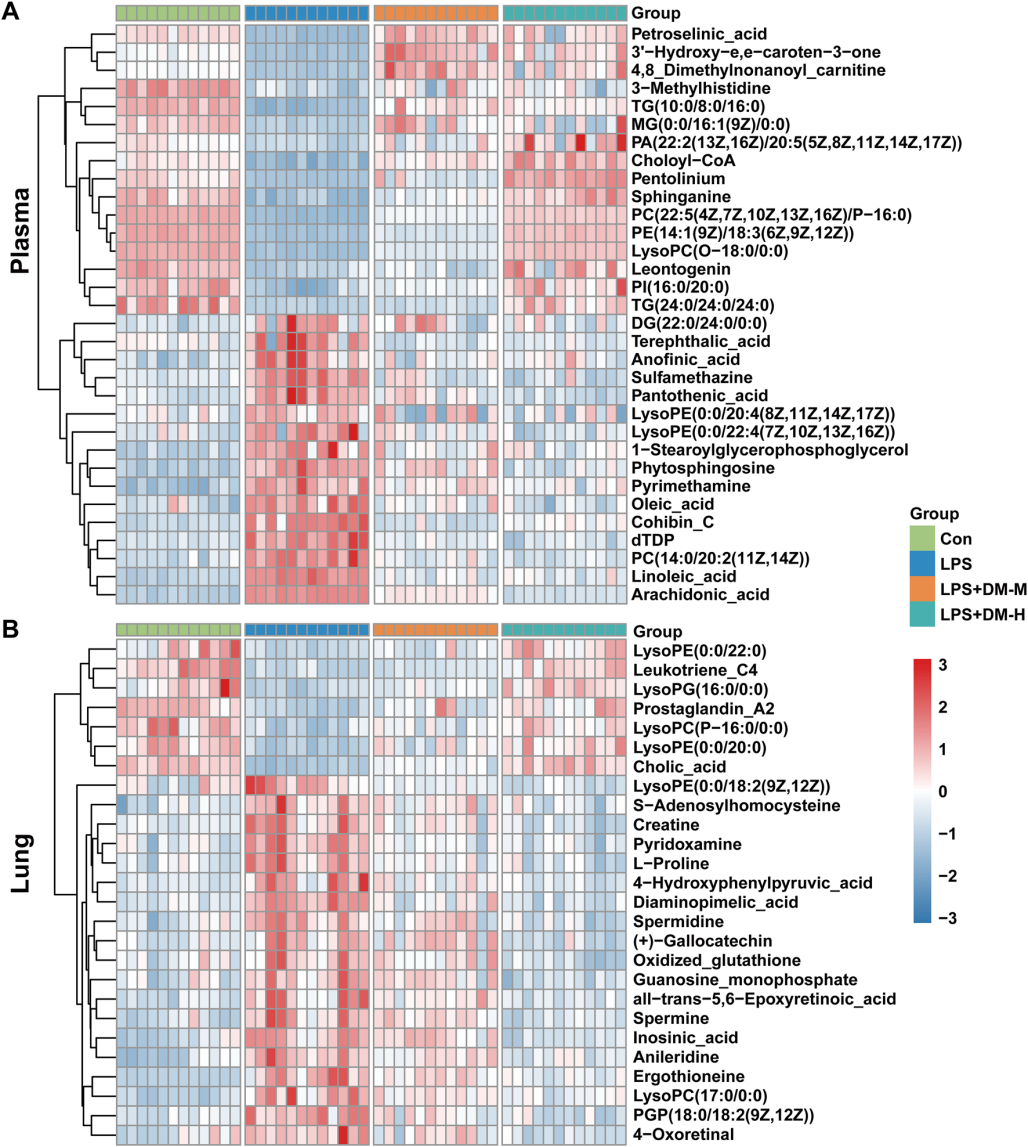
The expression of differential metabolites in mouse plasma (**A**) and lung tissue (**B**)

The differential metabolites were identified by HMDB, and 32 metabolites in the plasma (Table 1) and 26 metabolites in the lung tissues (Table 2) were screened. To observe the expression of differential metabolites in the four groups of samples, the differential metabolites were visualized with a heatmap analysis (Fig. 5A, B), in which the metabolites changed significantly between the control and LPS groups, and most of these were reversed in the DM treatment group.

**Table 1:** The differential metabolites in plasma of mice

NO.	Metabolites	Formula	Rt (min)	M/Z	VIP	Con vs. LPS		LPS vs.DM-M		LPS vs.DM-H		Fragmentations (m/z)
						P	FC	P	FC	P	FC	
1	PI(20:0/16:0)	C_45_H_87_O_13_P	17.262	866.3584	1.21076	5.05E-10	0.43788	2.42E-05	1.59392	3.64E-07	2.15291	255.2329; 627.3515
2	PE(14:1(9Z)/18:3(6Z,9Z,12Z))	C_37_H_66_NO_8_P	2.452	701.4933	3.70328	4.73E-26	0.39028	6.18E-15	1.48864	6.24E-23	2.34811	565.4227; 663.3996
3	Sphinganine	C_18_H_39_NO_2_	2.386	340.2589	1.26835	1.17E-12	0.37635	6.71E-08	1.84174	2.33E-11	2.69873	60.0443; 284.2973
4	PC(22:5(4Z,7Z,10Z,13Z,16Z)/P-16:0)	C_46_H_82_NO_7_P	2.506	814.5797	4.90904	1.68E-28	0.39893	1.52E-21	1.74587	9.63E-27	2.24652	184.0733; 792.5902
5	Choloyl-CoA	C_45_H_74_N_7_O_20_P_3_S	2.517	1148.7852	1.48302	3.66E-13	0.59524	4.62E-13	1.68328	8.87E-13	1.9727	1122.3784;1140.3889
6	LysoPC(O-18:0/0:0)	C_26_H_56_NO_6_P	2.64	510.3876	1.02389	4.10E-25	0.40162	3.97E-17	1.57946	9.81E-25	2.30119	104.0912; 510.3880
7	Phytosphingosine	C_18_H_39_NO_3_	4.147	318.3007	2.15063	2.22E-10	2.62828	0.00088	0.76484	1.16E-08	0.52956	60.0452; 318.3008
8	Pyrimethamine	C_12_H_13_C_1_lN_4_	5.389	248.1071	2.29407	6.02E-10	1.83568	0.00067	0.83932	1.74E-05	0.76639	198.0808; 233.0664
9	Sulfamethazine	C_12_H_14_N_4_O_2_S	7.174	279.0921	2.75416	1.94E-08	1.45655	7.71E-07	0.71174	6.10E-09	0.63607	124.0271; 149.0230
10	Pantothenic acid	C_9_H_17_NO_5_	7.348	219.1324	1.06118	2.19E-06	1.57678	0.00023	0.74936	6.16E-07	0.595687	90.0550; 220.1178
11	dTDP	C_10_H_16_N_2_O_11_P_2_	7.272	435.0549	1.00089	2.82E-08	2.1878	7.15E-08	0.52578	1.99E-09	0.441967	127.0586
12	Linoleic acid	C_18_H_32_O_2_	11.983	280.3053	1.65539	2.77E-16	2.74883	4.69E-11	0.52958	4.61E-10	0.53843	41.0810; 263.1666
13	PC(14:0/20:2(11Z,14Z))	C_42_H_80_NO_8_P	13.172	758.5703	2.46195	5.09E-10	1.9892	1.39E-08	0.59024	2.39E-09	0.52511	184.0732; 758.5725
14	3'-Hydroxy-e,e-caroten-3-one	C_40_H_54_O_2_	2.331	566.43	2.61367	0.000157	0.266704	0.019972	4.068175	3.50E-06	5.407536	287.1794; 349.2458
15	TG(10:0/8:0/16:0)	C_37_H_70_O_6_	7.152	679.5111	3.82137	6.23E-07	0.216653	0.533412	1.454168	0.000318	3.093924	127.1117; 611.5245
16	MG(0:0/16:1(9Z)/0:0)	C_19_H_36_O_4_	2.371	351.2506	1.18789	3.31E-07	0.088711	5.35E-05	11.18208	0.039996	5.920102	351.2506
17	4,8 Dimethylnonanoyl carnitine	C_18_H_35_NO_4_	2.435	352.2458	1.15007	1.70E-06	0.161729	0.037749	5.573445	0.102297	3.884997	85.0286; 330.2638
18	TG(24:0/24:0/24:0)	C_75_H_146_O_6_	2.527	1143.8027	1.9883	0.0043	0.680223	2.95E-09	3.449353	3.28E-08	4.221253	775.7538; 1144.1192
19	3-Methylhistidine	C_7_H_11_N_3_O_2_	5.389	867.1071	2.29407	0.001348	1.774902	0.054547	0.701298	0.009556	0.577705	865.5811
20	Terephthalic acid	C_8_H_6_O_4_	7.19	149.0231	1.94229	0.03816	1.556249	0.00322	0.444208	0.004775	0.47752	121.0295
21	Anofinic acid	C_12_H_12_O_3_	7.19	205.0859	1.49016	1.16E-05	2.118335	0.00057	0.63091	0.001171	0.665832	187.0754; 205.0859
22	1-Stearoylglycerophosphoglycerol	C_24_H_49_O_9_P	9.283	530.3452	2.62522	0.012386	2.746449	0.00105	0.597754	0.855365	1.057998	267.2682; 341.3050
23	DG(22:0/24:0/0:0)	C_49_H_96_O_5_	9.153	763.2448	1.08291	0.003817	2.208159	0.175033	0.71258	0.024067	0.57913	397.3676; 425.3989
24	Pentolinium	C_15_H_32_N_2_	9.578	263.2458	1.30865	0.00047	2.313994	0.004618	0.577774	0.001309	0.503244	96.0810; 1,140,920
25	Cohibin C	C_37_H_68_O_4_	15.859	577.519	1.06788	0.000343	15.15793	0.010844	0.391416	0.00119	0.180381	81.0355; 541.4979
26	PA(22:2(13Z,16Z)/20:5(5Z,8Z,11Z,14Z,17Z))	C_45_H_75_O_8_P	17.227	778.7852	1.840872	2.64E-05	0.406488	0.004533	2.07273	0.002669	3.610348	699.5323; 797.5092
27	Leontogenin	C_27_H_42_O_5_	17.217	464.3371	1.464973	2.50E-06	0.207219	0.096437	1.677627	0.045176	2.848881	4.0386; 429.2999
28	PI(16:0/20:0)	C_45_H_87_O_13_P	2.242	865.3286	1.815752	4.00E-06	0.325564	0.011288	2.267752	0.002873	5.618772	311.2955; 571.2889
29	LysoPE(0:0/20:4(8Z,11Z,14Z,17Z))	C_25_H_44_NO_7_P	6.098	524.2748	4.70183	0.000472	1.368666	0.055892	0.7098	0.002355	0.620224	44.0495; 361.2737
30	LysoPE(0:0/22:4(7Z,10Z,13Z,16Z))	C_27_H_48_NO_7_P	6.346	552.3022	1.5285	0.001548	1.62543	0.0153	0.72558	0.00071	0.57564	389.3050; 487.2819
31	Arachidonic acid	C_20_H_32_O_2_	10.543	327.2306	4.0233	2.86E-23	1.75205	7.40E-15	0.79066	4.25E-22	0.64154	303.2335
32	Oleic acid	C_18_H_34_O_2_	10.654	283.2625	1.01725	6.71E-07	1.82879	7.30E-06	0.66678	3.83E-08	0.53553	69.0000; 247.0245

**Table 2:** The differential metabolites in lung tissue of mice

NO.	Metabolites	Formula	Rt (min)	M/Z	VIP	Con vs. LPS		LPS vs.DM-M		LPS vs.DM-H		Fragmentations (m/z)
						P	FC	P	FC	P	FC	
1	Ergothioneine	C_9_H_15_N_3_O_2_S	0.94	230.0975	6.85519	8.89E-06	5.223586	0.028336	0.722204	0.000399	0.484999	69.0580; 127.0362
2	( +)-Gallocatechin	C_15_H_14_O_7_	1.07	307.0829	5.01184	3.85E-14	1.539765	2.77E-09	0.776547	9.53E-09	0.573716	139.0390; 307.08113
3	Oxidized glutathione	C_20_H_32_N_6_O_12_S_2_	1.08	635.142	1.24098	0.001474	1.349826	0.063388	0.857859	1.44E-05	0.603938	355.0759; 484.1187
4	Inosinic acid	C_10_H_13_N_4_O_8_P	0.97	349.056	3.46688	1.50E-06	2.506744	0.06727	0.82939	1.75E-06	0.426869	97.0298; 137.0457
5	Guanosine monophosphate	C_10_H_14_N_5_O_8_P	0.95	364.0649	3.05321	0.00017	1.608101	0.084982	0.856493	4.66E-06	0.533426	152.0562
6	S-Adenosylhomocysteine	C_14_H_20_N_6_O_5_S	1.05	385.1303	2.64985	2.61E-05	1.629015	0.016513	0.811106	0.00022	0.686493	112.0183; 170.0849
7	all-trans-5,6-Epoxyretinoic acid	C_20_H_28_O_3_	13.52	317.2117	2.30797	0.000264	1.933585	0.045855	0.778557	0.000488	0.550082	79.0542; 281.1900
8	Creatine	C_4_H_9_N_3_O_2_	0.91	132.0775	1.79766	6.98E-05	1.485564	0.002392	0.766444	5.19E-06	0.595529	44.1000; 55.2000
9	LysoPC(17:0/0:0)	C_25_H_52_NO_7_P	18.32	527.3804	1.36725	7.89E-06	3.681324	0.018519	0.721019	0.000698	0.545856	184.0733; 492.3449
10	Pyridoxamine	C_8_H_12_N_2_O_2_	0.78	191.0781	1.1411	1.63E-06	1.624016	3.72E-06	0.662852	5.36E-07	0.599696	134.0606; 151.0871
11	Spermidine	C_7_H_19_N_3_	0.72	146.1654	1.13866	1.21E-05	1.445056	0.007625	0.818006	2.44E-06	0.659883	72.0820; 84.0840
12	L-Proline	C_5_H_9_NO_2_	0.91	116.0703	1.06339	3.29E-06	1.593842	4.40E-05	0.70307	3.14E-06	0.605171	70.0651; 41.0386
13	Spermine	C_10_H_26_N_4_	13.76	241.18	1.05343	4.59E-05	2.891314	0.077649	0.785466	8.89E-05	0.389801	58.0657; 186.1970
14	4-Hydroxyphenylpyruvic acid	C_9_H_8_O_4_	3.68	181.0491	1.19548	0.000492	2.762776	0.011312	0.58101	0.000701	0.392195	77.0391; 107.0497
15	Diaminopimelic acid	C_7_H_14_N_2_O_4_	0.79	213.0852	1.8311	1.40E-09	2.180373	9.35E-06	0.565259	7.44E-10	0.411171	82.0652; 128.0705
16	LysoPC(P-16:0/0:0)	C_24_H_50_NO_6_P	19	480.3413	1.87704	1.64E-06	0.514088	0.003854	1.339187	3.65E-06	1.649673	180.0188; 462.3343
17	Leukotriene C4	C_30_H_47_N_3_O_9_S	12.14	626.3128	1.31377	7.35E-08	0.286374	0.024872	1.351636	3.93E-07	2.82934	189.1638; 319.2268
18	LysoPE(0:0/18:2(9Z,12Z))	C_23_H_44_NO_7_P	16.79	478.2935	1.615482	0.002276	1.730487	0.000351	0.530287	2.76E-05	0.393158	263.2369; 337.2737
19	PGP(18:0/18:2(9Z,12Z))	C_42_H_80_O_13_P_2_	29.14	872.5415	1.854269	3.27E-06	2.225789	0.000646	0.628777	1.16E-06	0.394302	603.5347; 757.5378
20	Anileridine	C_22_H_28_N_2_O_2_	18.7	353.2236	1.5043	4.90E-07	2.232817	0.004675	0.783746	1.08E-05	0.600209	120.0808; 279.1856
21	4-Oxoretinal	C_20_H_26_O_2_	13.32	299.2014	1.25101	0.000157	1.724575	0.008411	0.73029	3.73E-06	0.487338	137.0961; 189.1274
22	LysoPE(0:0/20:0)	C_25_H_52_NO_7_P	19.76	510.3511	2.43664	9.46E-09	0.428477	0.000292	1.721601	4.79E-07	2.180142	44.0495; 369.3363
23	LysoPE(0:0/22:0)	C_27_H_56_NO_7_P	23.79	538.3835	1.752036	0.000226	0.435181	0.009919	1.432045	3.59E-06	2.272196	44.0495; 397.3676
24	LysoPG(16:0/0:0)	C_22_H_45_O_9_P	21.28	483.2727	4.0505	0.000257	0.438891	2.68E-05	1.515575	2.20E-08	2.256267	483.2728
25	Cholic acid	C_24_H_40_O_5_	13.08	407.2826	2.24678	1.49E-14	0.315424	2.92E-09	2.107035	3.85E-10	3.146423	345.2794; 373.2743
26	Prostaglandin A2	C_20_H_30_O_4_	14.39	333.2069	1.57342	1.37E-11	0.391936	0.004173	1.707462	4.52E-06	2.156729	67.0542; 299.2006

**Fig. 6:**
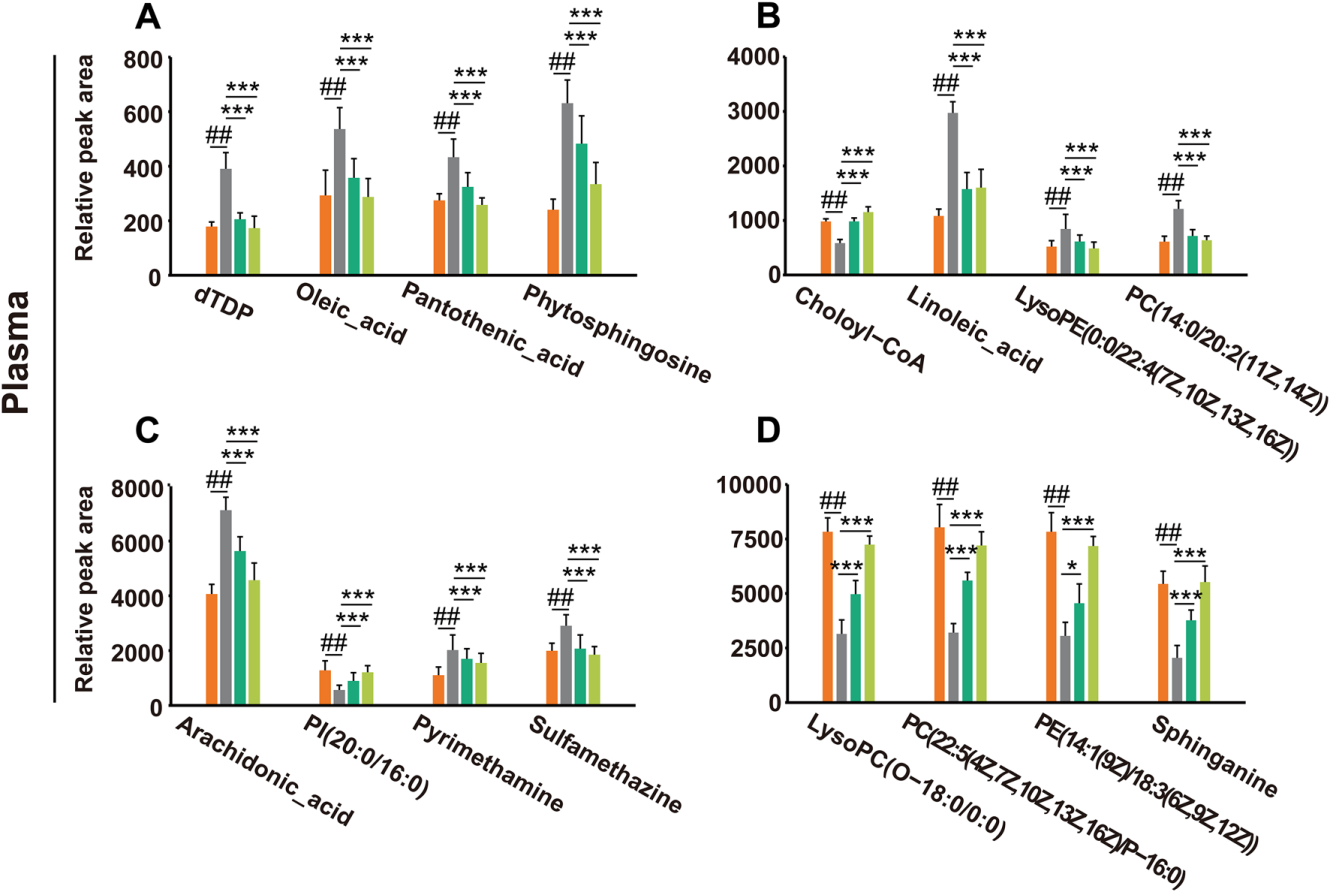
The ionic strength of potential metabolites in mouse plasma (**A**–**D**). Data are displayed as Mean ± SD. # *P* < 0.05, ## *P* < 0.01 vs. control group, *P < 0.05, **P < 0.01 vs. LPS group

**Fig. 7:**
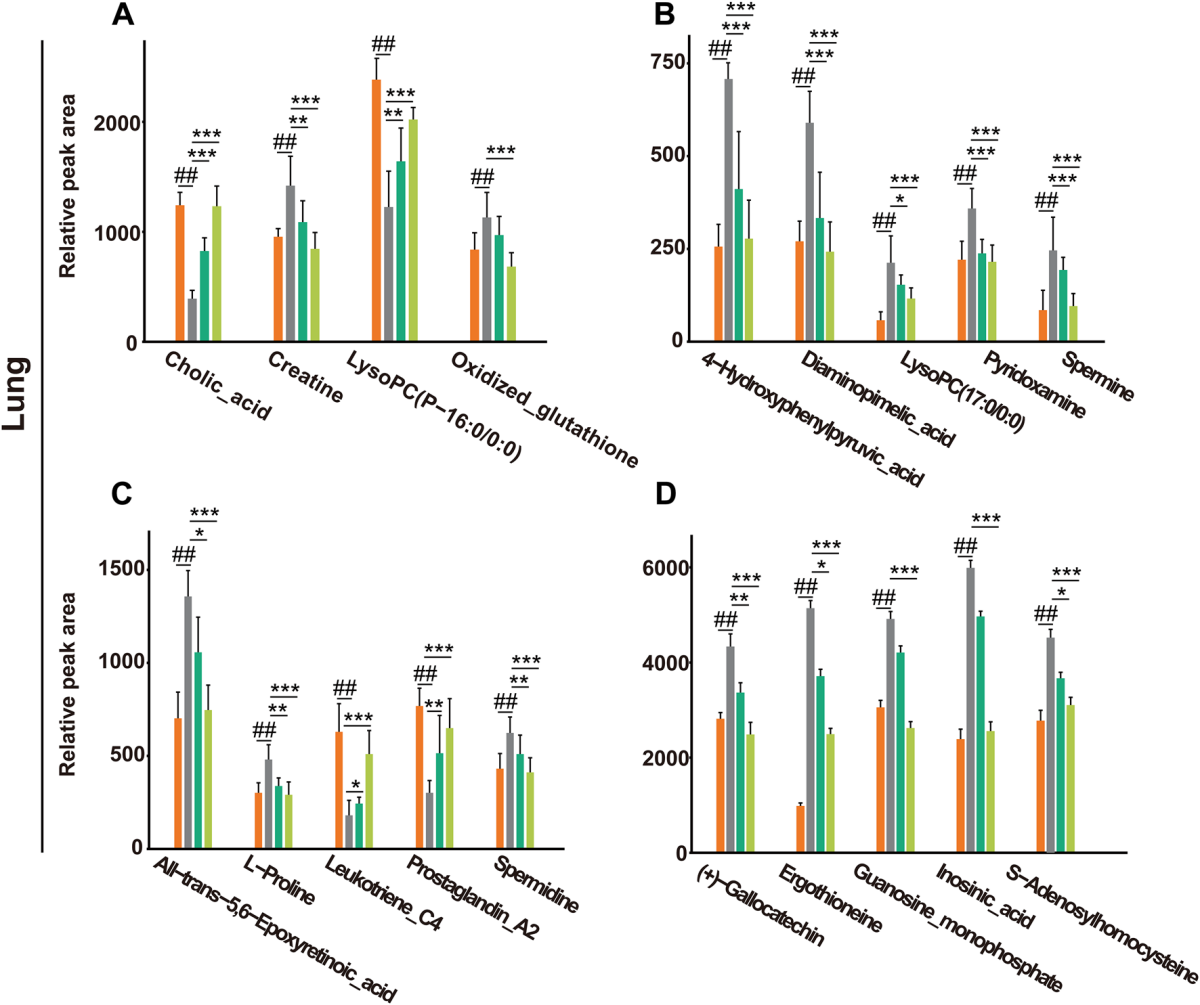
The ionic strength of potential metabolites in mouse lung tissues (**A**–**D**). Data are displayed as Mean ± SD. #*P* < 0.05, ##*P* < 0.01 vs. control group, *P < 0.05, **P < 0.01 vs. LPS group

A total of 16 potential metabolites in mouse plasma samples were identified (Fig. 6A–D). Compared with the control group, phytosphingosine, pyrimethamine, sulfamethazine, pantothenic acid, dTDP, linoleic acid, PC(14:0/20:2(11Z,14Z)), LysoPE (0:0/22:4(7Z,10Z,13Z,16Z)), arachidonic acid and oleic acid were significantly upregulated in the LPS group, whereas sphinganine, Choloyl-CoA, PI(20:0/16:0), PE(14:1(9Z)/18:3(6Z,9Z,12Z)), PC(22:5(4Z,7Z,10Z,13Z,16Z)/P-16:0), and LysoPC (O-18:0/0:0) were downregulated. Moreover, as shown in Fig. 7A–D, a total of 19 potential metabolites in the lung tissues were identified in which ergothioneine, ( +)-gallocatechin, oxidized glutathione, inosinic acid, guanosine monophosphate, S-adenosylhomocysteine, all-trans-5,6-epoxyretinoic acid, creatine, lysoPC(17:0/0:0), pyridoxamine, spermidine, L-proline, spermine, 4-hydroxyphenylpyruvic acid, and diaminopimelic acid were upregulated in the LPS group compared with those in the control group, and lysoPC (P-16:0/0:0), leukotriene C4, cholic acid and prostaglandin A2 were downregulated. After treatment with DM, the levels of these potential metabolites returned to normal, which indicated that DM treatment could effectively improve the metabolic disorder of ALI mice.

### Metabolic pathway analysis

**Fig. 8:**
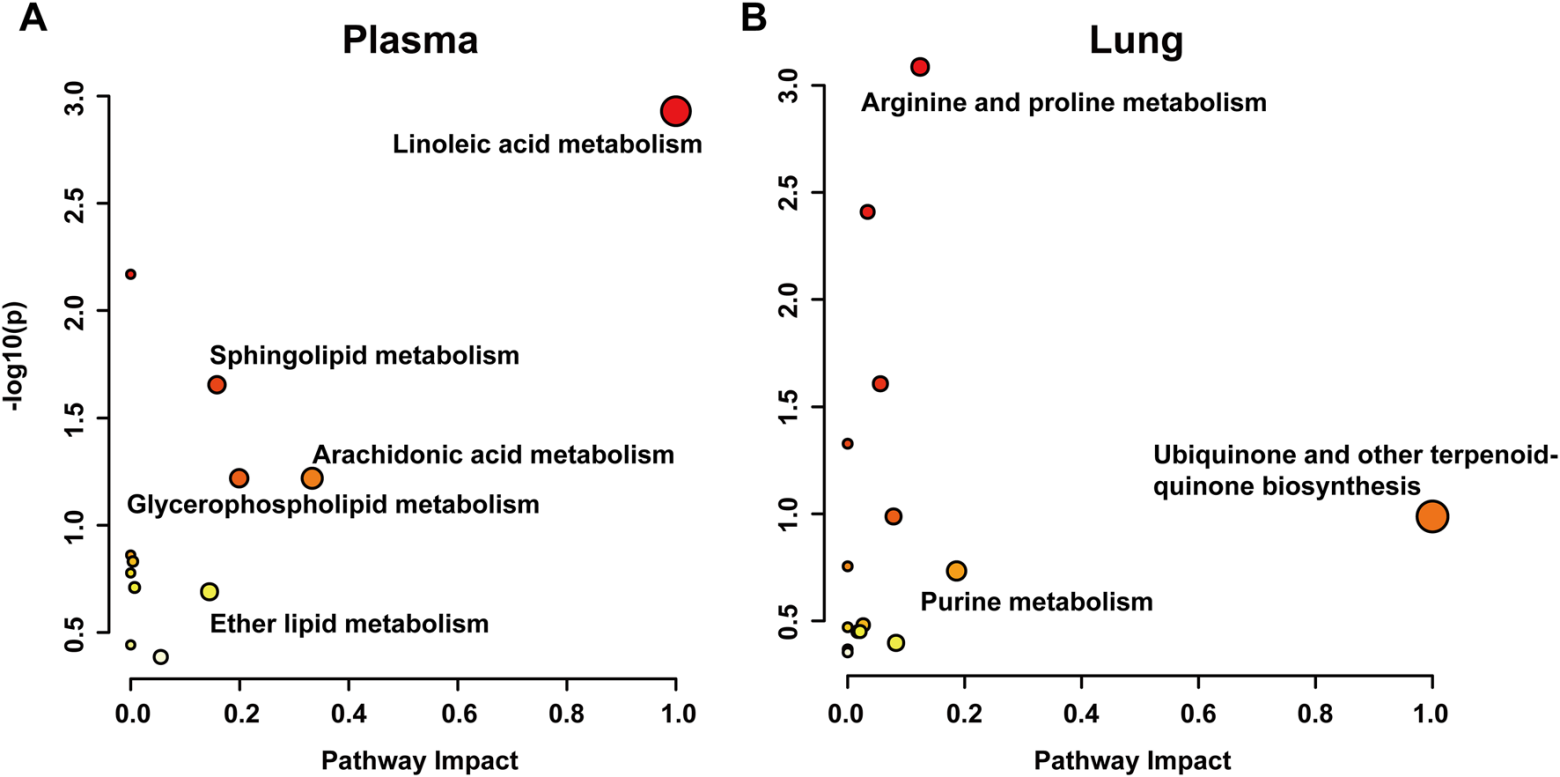
Enrichment analysis of potential metabolic pathways in mouse plasma (**A**) and lung tissue (**B**)

The candidate metabolites were imported into the MetaboAnalyst database for metabolic pathway enrichment analysis, and 12 metabolic pathways were found in plasma and 15 were found in lung tissue, among which glycerophospholipid metabolism, arachidonic acid metabolism, and primary bile acid biosynthesis were shared in mouse plasma and lung tissue (Additional file 2: Table S2). Based on a pathway impact > 0.1, linoleic acid metabolism, arachidonic acid metabolism, glycerophospholipid metabolism, sphingolipid metabolism, and ether lipid metabolism were significantly affected in the plasma (Fig. 8A). Moreover, ubiquinone and other terpenoid quinone biosynthesis, purine metabolism, and arginine and proline metabolism were notably affected in the lung tissue (Fig. 8B).

### Network pharmacology analysis

**Fig. 9:**
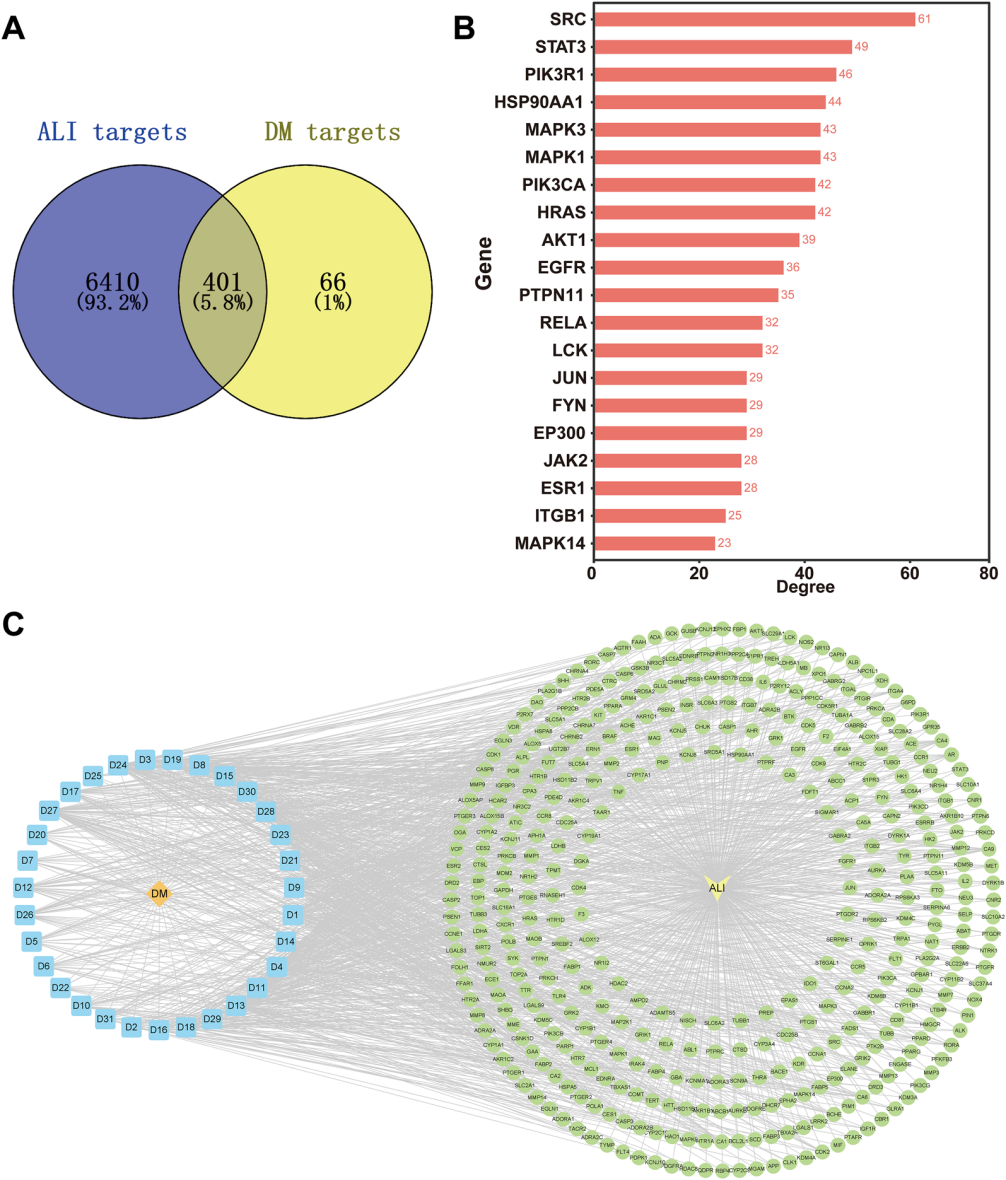
Network pharmacological analysis of DM in the treatment of ALI. **A** Shared targets of DM and ALI. **B** Core genes for DM treatment of ALI. **C** “DM- active components -target-ALI” network, orange: DM, blue: active components (31), yellow: ALI, green: shared target (401)

To further explore the mechanism of DM against ALI, we performed a network pharmacology analysis. First, we searched the literature for 166 chemical constituents in DM, mainly alkaloids, phenolic acids, glycosides, terpenoids, flavonoids, and other compounds (Additional file 3: Table S3). Next, 467 targets related to 31 active components of DM were collected from BATMAN-TCM, DrugBank and Swiss Target Prediction. Subsequently, 6811 related targets of ALI were obtained from the DrugBank, DisGeNET, OMIM, and TTD databases. Ultimately, 401 targets related to DM and ALI were identified as potential targets of DM to treat ALI (Fig. 9A).

To obtain the protein–protein interaction relationship between DM active components and ALI targets, the 401 targets were imported into the STRING database for PPI network analysis, and the top 20 core genes were screened (Fig. 9B). The common targets of DM active components and ALI were imported into Cytoscape 3.9.1 software to construct the “DM-active ingredient-target-ALI” network. Figure 9C shows the 31 active components and 401 potential targets of DM in the treatment of ALI, and the importance of active components was evaluated by degree, betweenness, and closeness in the network (Additional file 3: Table S4).

**Fig. 10:**
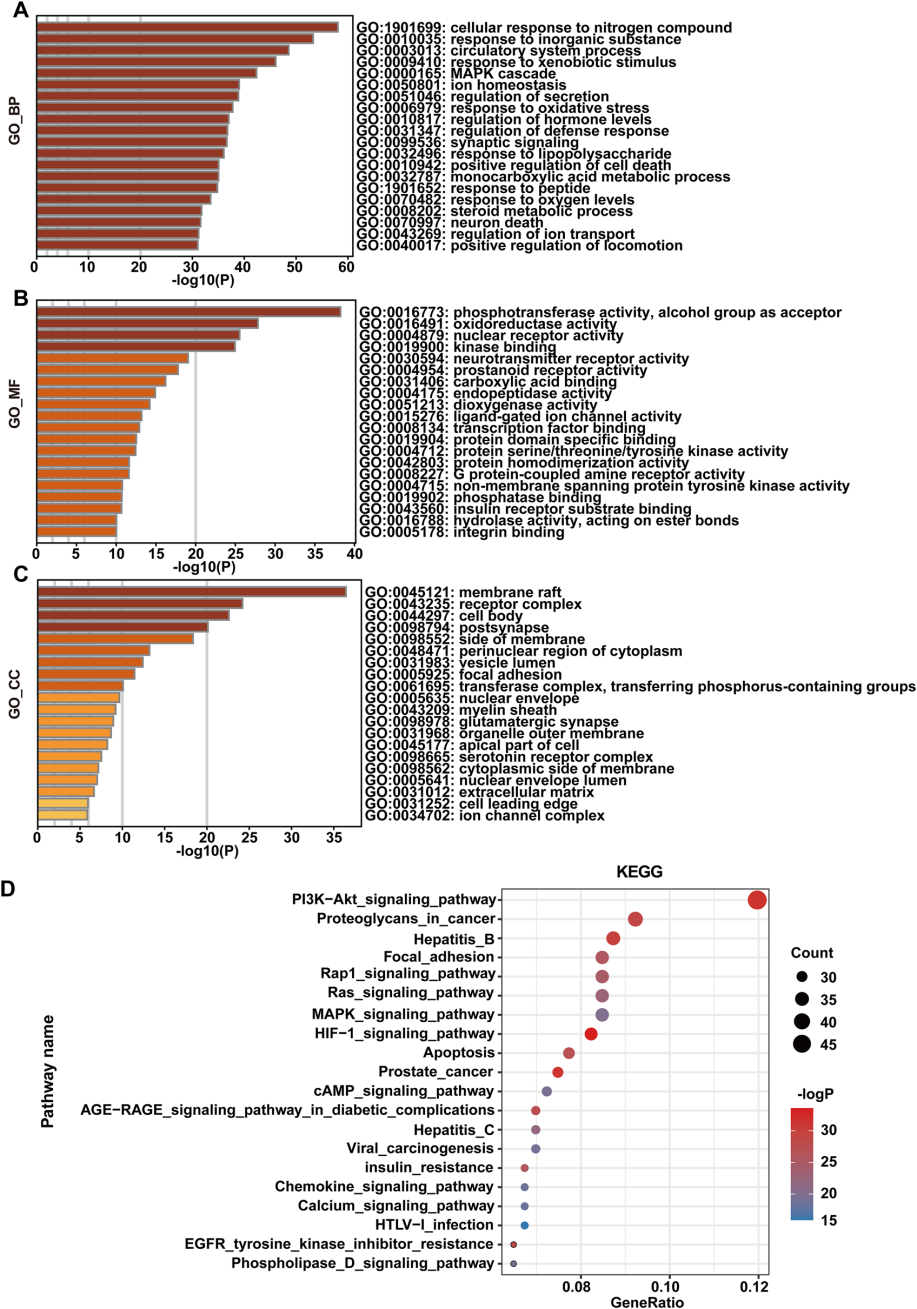
Enrichment analysis of GO and KEGG pathways. **A** biological process **B** molecular function **C** cell composition **D** KEGG enrichment analysis

To predict the therapeutic mechanism of DM on ALI, we carried out GO and KEGG enrichment analyses, in which the GO enrichment analysis included biological processes (BP), cellular components (CC) and molecular functions (MF), which explained the biological function of genes at different levels. Biological processes mainly included cellular response to nitrogen compounds, the MAPK cascade, response to oxidative stress, etc. (Fig. 10A). Molecular functions mainly included phosphotransferase activity, the alcohol group as acceptor, oxidoreductase activity, nuclear receptor activity, etc. (Fig. 10B). The cell components mainly included the membrane raft, receptor complex, cell body, etc. (Fig. 10C). According to the KEGG enrichment analysis, 20 pathways were significantly affected, including the PI3K-Akt signaling pathway, MAPK signaling pathway, HIF-1 signaling pathway, and apoptosis, of which PI3K/Akt was identified as a potential pathway for DM to treat ALI (Fig. 10D).

### Comprehensive analysis of metabolomics and network pharmacology

**Fig. 11:**
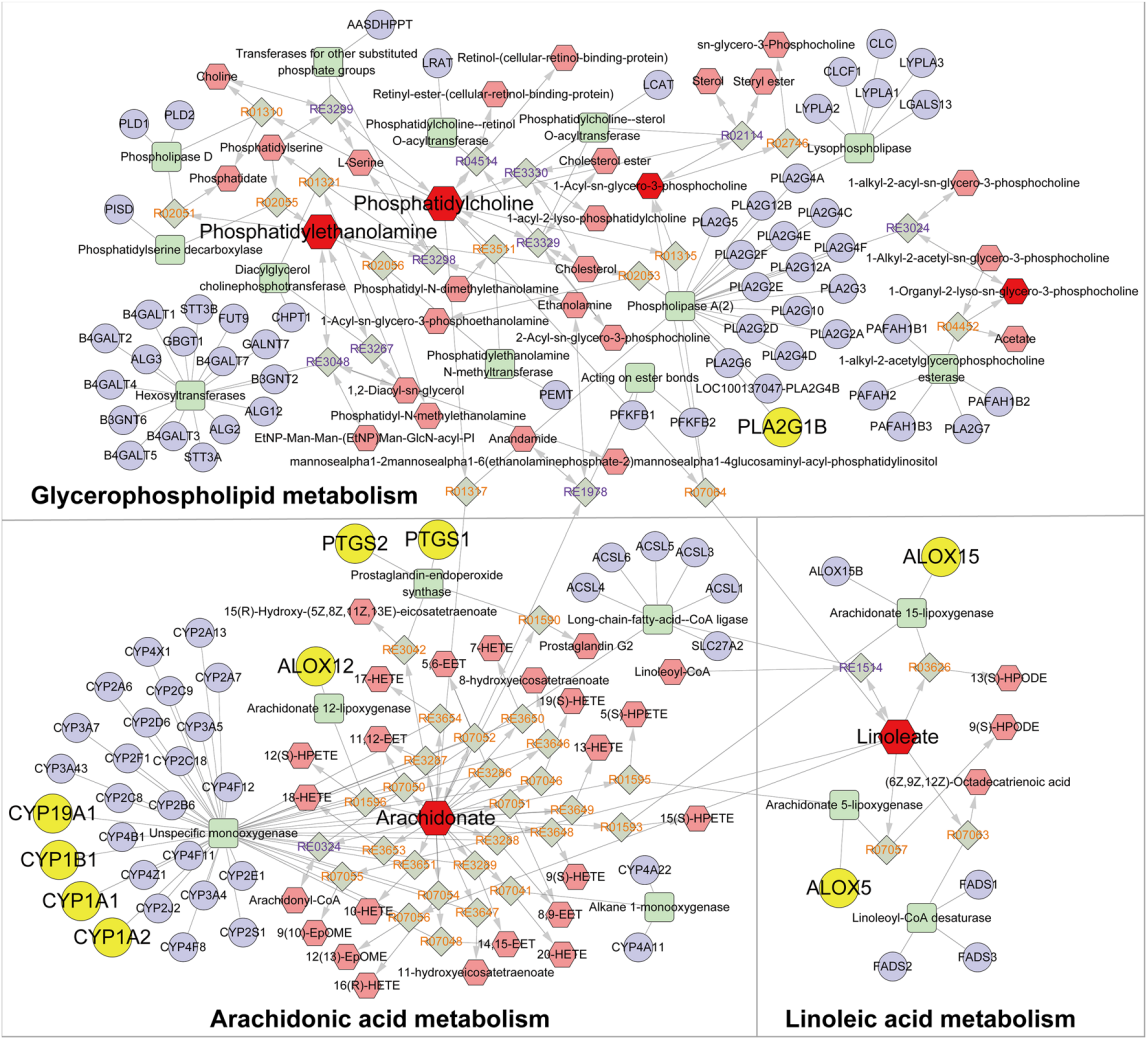
“Potential metabolite-reaction-enzyme-gene” interaction network. Red hexagons represent metabolites, gray diamonds represent reactions, green rectangles represent enzymes, and purple circles represent genes

Metabolomics and network pharmacology were integrated to construct an interaction network. First, we introduced candidate metabolites from plasma and lung tissues into MetScape to construct a “Metabolite-Reaction-Enzyme-Gene” network (Fig. 11). Additionally, we matched the genes associated with the candidate metabolites with the potential targets of network pharmacology and found 10 key targets, including PTGS2, PLA2G1B, CYP1A2, ALOX15, PTGS1, ALOX5, ALOX12, CYP1A1, CYP1B1, and CYP19A1 (Additional file 5: Fig. S7). The affected pathways were arachidonic acid metabolism, linoleic acid metabolism, and glycerophospholipid metabolism. These pathways may be involved in inflammation and cell apoptosis.

### Biological experimental verification

To evaluate the accuracy of the method and results of the integrated analysis of network pharmacology and metabolomics, we used immunohistochemistry and western blot methods to determine the proteins (PI3K/Akt and Bax/Bcl-2) associated with inflammation and cell apoptosis as validation.

**Fig. 12:**
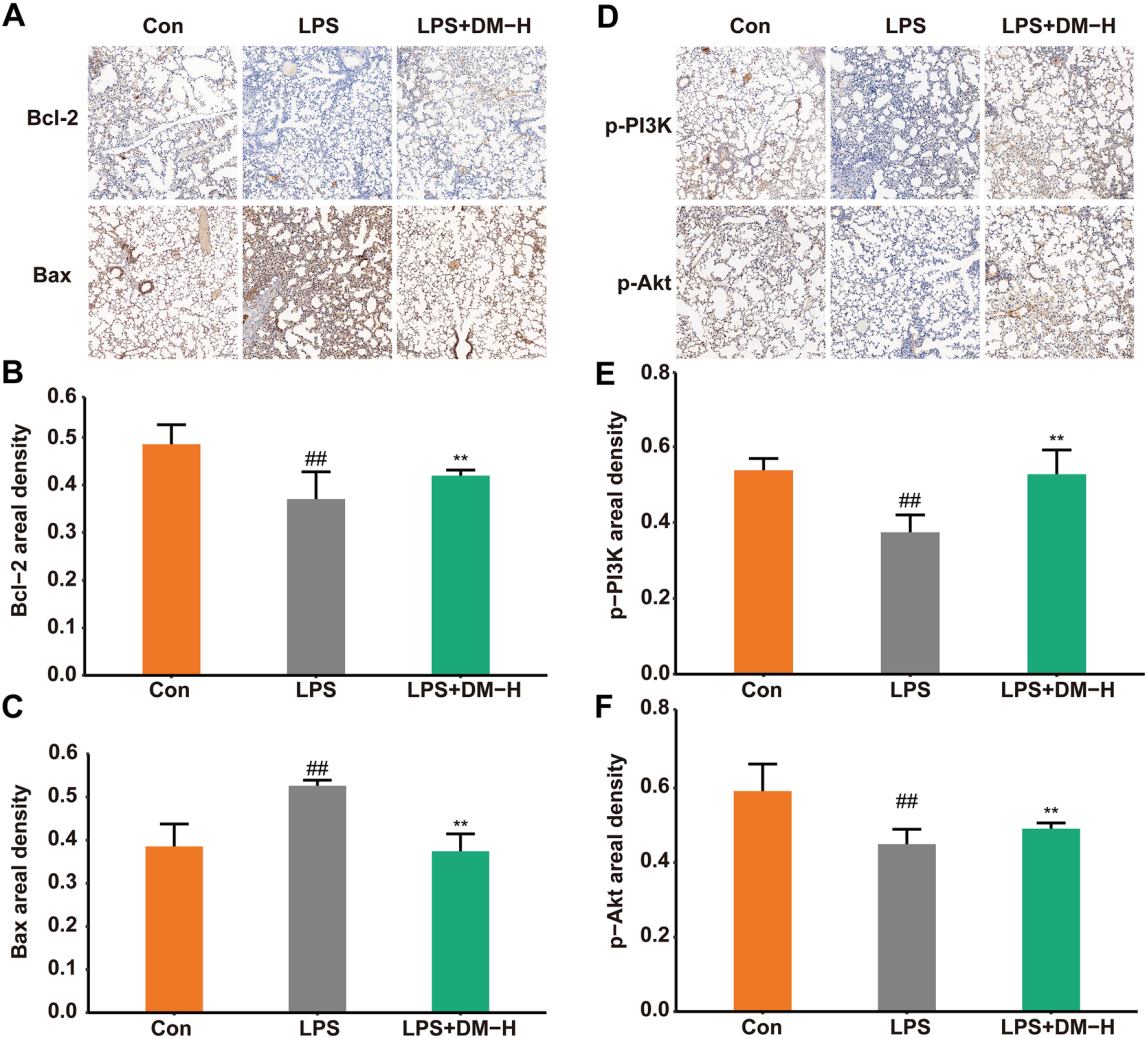
Cell proliferation and apoptosis detection of LPS-induced ALI. **A** Immunohistochemistry staining of Bcl-2 and Bax proteins (magnification 200 ×). The expression of Bcl-2 (**B**) and Bax (**C**) in lung tissue. **D** Immunohistochemistry staining of p-PI3K and p-Akt proteins (magnification 200 ×). The expression of p-PI3K (**E**) and p-Akt (**F**) in lung tissue. Data are displayed as Mean ± SD. #*P* < 0.05, ##*P* < 0.01 vs. control group, *P < 0.05, **P < 0.01 vs. LPS group

Immunohistochemistry was used to investigate the effect of DM on LPS-induced ALI mouse lung tissue apoptosis. As shown in Fig. 12A–C, the expression of Bcl-2 was downregulated, and Bax was upregulated after LPS stimulation. However, after DM treatment, the expression of Bcl-2 was promoted, and Bax was inhibited, indicating that DM may have an antiapoptotic effect on LPS-induced ALI mice. The PI3K/Akt pathway regulates cell proliferation, apoptosis, and the release of anti-inflammatory cytokines. Therefore, we also examined the phosphorylation levels of PI3K and Akt in the lung tissue of ALI mice, which were downregulated after LPS induction but were significantly reversed by DM treatment (Fig. 12D–F). It was suggested that DM might have a therapeutic effect on ALI by activating PI3K/Akt.

**Fig. 13:**
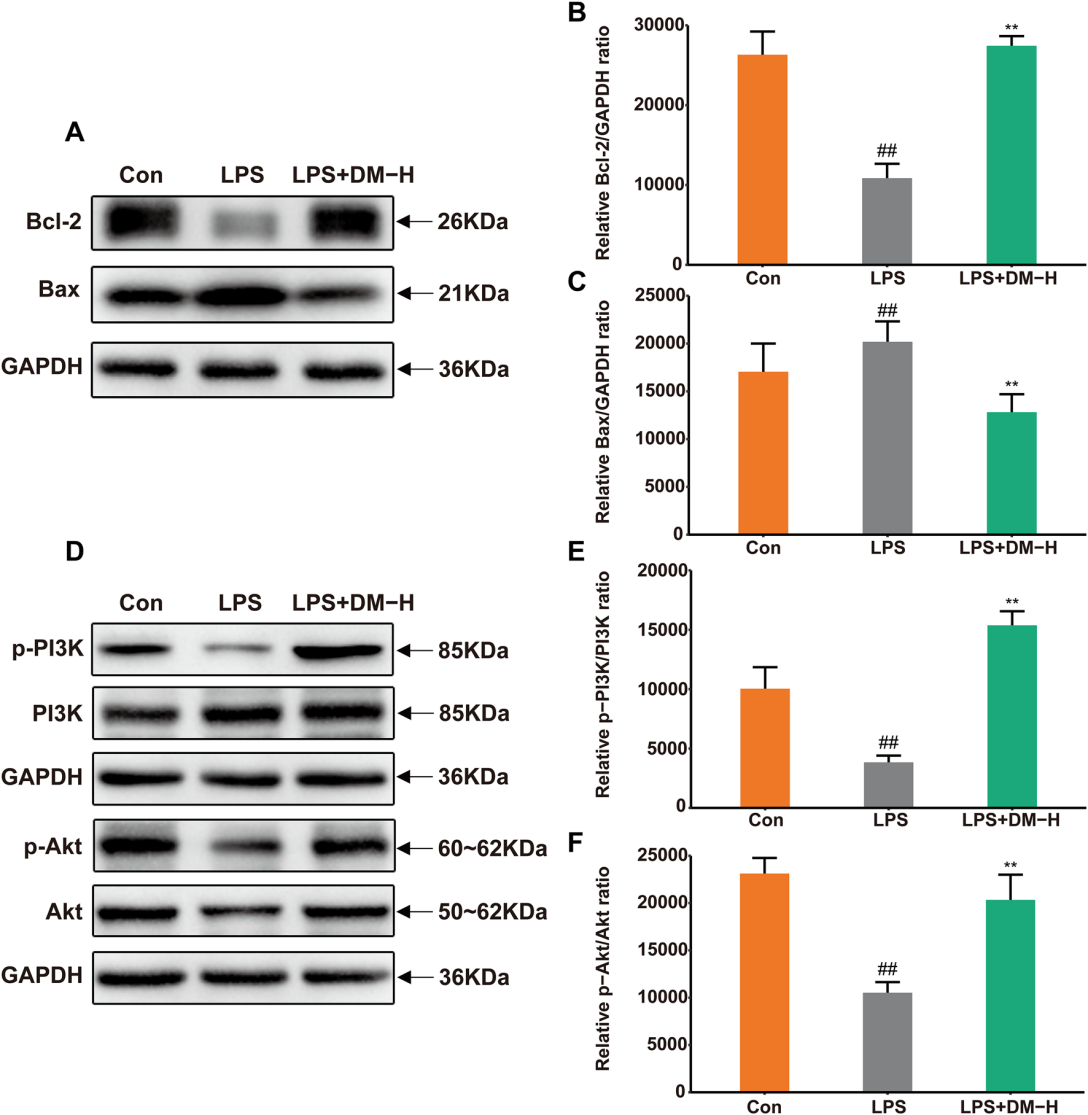
The proliferation and apoptosis of ALI cells were detected by western blotting. **A** Images of Bcl-2 and Bax proteins. The expression of Bcl-2 (**B**) and Bax (**C**) in lung tissue. **D** Images of p-PI3K and p-Akt proteins. The expression of p-PI3K **E** and p-Akt **F** in lung tissue. Data are displayed as the mean ± SD. #*P* < 0.05, ##*P* < 0.01 vs. control group and *P < 0.05, **P < 0.01 vs. LPS group

To further verify whether DM could regulate apoptosis in LPS-induced ALI mice, we detected the levels of Bcl-2 and Bax in the lung tissue of ALI mice by western blotting. After LPS stimulation, the expression of Bcl-2 was downregulated, and Bax was significantly upregulated. After DM treatment, the level of Bcl-2 was significantly increased, and Bax was significantly decreased (Fig. 13A–C). The results revealed that DM could inhibit LPS-induced apoptosis. Furthermore, we also detected the expression of PI3K/Akt in lung tissue. LPS caused a decrease in the phosphorylation levels of PI3K and Akt, whereas DM treatment significantly increased their expression (Fig. 13D–F). These data confirm that DM could effectively ameliorate LPS-induced ALI by activating PI3K/Akt.

## Discussion

*Nauclea officinalis* is involved in the regulation of the inflammatory response, oxidative stress, immune regulation, and cell proliferation [29]. These activities are closely related to the occurrence and development of ALI. Previous studies have demonstrated that DM could inhibit LPS-induced secretion of NO, TNF-α, IL-1β, and IL-6 and inhibit the phosphorylation levels of NF-κ B, p38, ERK, and JNK, showing outstanding anti-inflammatory activity [30]. In this study, we found that DM could maintain the structural integrity of lung tissue in ALI mice, specifically by inhibiting the infiltration of inflammatory cells and improving alveolar wall thickening, alveolar collapse, and hemorrhagic characteristics. These results reveal that DM could effectively improve lung injury.

In the metabolomic analysis, samples of diseased organs are typically collected for subsequent analysis. From a physiological point of view, the lung is the main diseased organ, and the blood is rich in small molecule metabolites. Changes in blood components can reflect the physiological and pathological changes of the organ. When the body is diseased, the inflammatory mediators in the blood are transferred to the lungs and attack lung tissue. Hence, this experiment chose blood and lung tissues as ideal specimens for metabolic analysis. We employed UPLC-QTOF-MS/MS to characterize and analyze endogenous metabolites in a DM-treated mouse model of LPS-induced ALI and found 16 candidate metabolites in plasma and 19 candidate metabolites in lung tissue. These metabolites are mainly related to metabolic pathways such as linoleic acid metabolism, arachidonic acid metabolism, glycerophospholipid metabolism, sphingolipid metabolism, purine metabolism, and arginine and proline metabolism.

Fatty acid metabolism is an essential component of biofilms and can supply energy for the body and play a prominent role in inhibiting inflammation, resisting bacterial and viral infections, and immune regulation [31]. Linoleic acid (LA) cannot be synthesized in the body and is an essential fatty acid. Moreover, LA produces arachidonic acid (AA), which can produce prostaglandins (PGs) and leukotrienes (LTs) under the action of cyclooxygenase (COX). Studies have shown that increasing LA intake may decrease the risk of pneumonia [32]. Moreover, AA has activity against bacteria, fungi, and influenza viruses, whereas alveolar macrophages can release AA and other unsaturated fatty acids into alveolar fluid, thereby exerting their antibacterial impact and protecting the lungs from various microbial infections [33]. The content of unsaturated fatty acids in ALI may change with the pathological state. One study found that in the LPS-induced ALI mouse model, LA had different trend changes in serum, lung, BALF and spleen [14]. Similarly, our study also demonstrated that the contents of LA and AA in the plasma of the ALI model group were increased, whereas the contents of PGA2 and LTC4 in the lung tissues were decreased. The conversion of excess LA to AA indicated that ALI mice were in an inflammatory state, and downregulation of PGA2 and LTC4 in lung tissues may be associated with changes in COX activity in disease states. Although PGs are proinflammatory factors, they have both pro- and anti-inflammatory effects. PGs may trigger the inflammatory process in the early stage of the disease. However, when the concentration of PGs reaches a sufficient level and the inflammatory process is at an optimal level, the anti-inflammatory process is initiated by increasing the synthesis of active anti-inflammatory lipids [34, 35]. In addition, other fatty acids, such as pantothenic acid and oleic acid, were observed to have increased in the mouse plasma samples of the model group. Pantothenic acid has anti-lipid peroxidation effects [36], whereas oleic acid plays a role in the inflammatory response [37]. According to reports, fatty acids can protect the body from disease by regulating various inflammatory cells, and cytokines can promote the production of fatty acids [38]. Some fatty acids can also inhibit apoptosis and reduce oxidative stress [39]. Additionally, we also found that the specific amount and type of glycerophospholipid changes in LPS-induced ALI mice differed in plasma and lung tissue samples [15]. Disturbances in glycerophospholipid metabolism are mainly affected by the activity of phospholipase A2, which in turn is associated with inflammation [40].

Sphingomyelin is involved in numerous biological processes such as cell growth and differentiation, regulation of membrane stability, apoptosis, and angiogenesis. Furthermore, studies have indicated that sphingomyelin degradation products are involved in the pathophysiological process of ALI [41]. Sphingomyelin regulates PI3K/Akt to improve vascular endothelial permeability, reduce vascular inflammation, and maintain vascular endothelial integrity [42]. Sphingosine is an essential mediator of pulmonary edema and alveolar damage, and it maintains alveolar stability by altering lung-damaging surfactants [31]. Our results showed that the levels of sphingosine in ALI mouse plasma were decreased, but the sphingosine levels were reversed after DM treatment. These data suggested that DM could activate sphingosine in LPS-induced ALI. Furthermore, arginine is involved in tissue repair, inflammation, and the secretion of various hormones [43]. It is a precursor for synthesizing nitric oxide, urea, polyamines, proline, glutamate, and creatine [44]. Elevation of proline in LPS-induced ALI results in increased neutrophil recruitment and protein leakage, suggesting that it mediates lung inflammation in ALI [45]. In our study, the L-proline levels in ALI mouse lung tissue were increased, and the expression was downregulated after treatment with DM, indicating that DM could alleviate ALI by inhibiting the increase in neutrophil recruitment and protein leakage during lung inflammation.

Our study identified 31 potentially active components of DM against ALI using network pharmacology analysis, among which strictosamide is the most abundant indole alkaloid in DM [8]. Strictosamide has excellent anti-inflammatory and analgesic activities, and its anti-inflammatory effect is related to the inhibition of the NF-κ B and MAPK signaling pathways [46]. Our previous study also indicated that strictosamide has favorable proliferation activity and angiogenesis effects on HUVECs. In this study, we found 20 core genes for DM treatment of ALI, which were mainly involved in cell growth, proliferation, differentiation and apoptosis; angiogenesis; and inflammatory responses. STAT3 can stimulate various growth factors, such as IFN, EGF, IL5, and IL6, and play a role in cell growth and apoptosis. However, this gene is also involved in coronavirus biology, immune response, and antiviral activity. Previous studies have shown that LPS-induced ALI can be improved by regulating the MAPK and IL-6/STAT3 signaling pathways [47, 48]. The MAPK kinase family is involved in various cellular processes such as cell proliferation, differentiation, and cell cycle progression. Studies have also shown that regulation of caspase-3, Bax/Bcl-2 and MAPK signaling could attenuate microvascular endothelial cell injury and alleviate inflammation and pulmonary edema in ALI mice while maintaining the integrity of alveolar structures [49]. Moreover, PI3K/Akt is involved in multifarious biological processes, including the regulation of cell proliferation, apoptosis, and the release of anti-inflammatory cytokines [50]. Our preceding studies have also confirmed that DM might repair the endothelial cell barrier of lung injury by activating the PI3K/Akt signaling pathway, inhibiting the inflammatory response, improving intercellular connections, and promoting angiogenesis. Therefore, it was determined that PI3K/Akt is the main influencing pathway of DM in the treatment of ALI.

The integrated analysis of metabolomics and network pharmacology found 10 key targets (PTGS2, PLA2G1B, CYP1A2, ALOX15, PTGS1, ALOX5, ALOX12, CYP1A1, CYP1B1, and CYP19A1) and 3 related pathways (arachidonic acid metabolism, linoleic acid metabolism, and glycerophospholipid metabolism). This analytical strategy is also suitable for screening metabolites and targets in other natural compounds. PTGS2, also known as cyclooxygenase, can catalyze AA to generate PGs, which are mainly involved in inflammation, cell proliferation and apoptosis. In addition, PTGS1 can regulate angiogenesis in endothelial cells [51]. PLA2G1B is a phospholipase A2 that catalyzes membrane glycerophospholipids to release AA and Lys phospholipids, which are involved in cell contraction and proliferation. The hydrolysis of surfactant phospholipids (PL) by phospholipase A2 results in surfactant damage in ALI/ARDS [52]. ALOX15, ALOX5, and ALOX12 are all members of the lipoxygenase protein family that can act on AA and LA and affect inflammation, immunity, cell proliferation, apoptosis, and angiogenesis [53]. CYP1A1, CYP1A2, CYP1B1, and CYP19A1 are members of the cytochrome P450 family and have a therapeutic effect on hyperoxia-induced lung injury [54]. After LPS stimulation, the levels of the AA-related genes CBR2, CYP4F18 and CYP2E1 were downregulated, whereas ALOX12, PTGES and PTGES2 were upregulated. Studies have shown that LPS-induced ALI can be alleviated by regulating AA, anti-apoptotic and anti-inflammatory effects [33]. In brief, integrated network pharmacology and metabolomic studies revealed that the effect of DM on reversing ALI is mainly mediated by regulating arachidonic acid metabolism, linoleic acid metabolism, and glycerophospholipid metabolism. Furthermore, these actions might be closely related to the regulation of the inflammatory response, cell proliferation and apoptosis.

Apoptosis of lung cells plays a pivotal role in ALI/ARDS development [55, 56]. Therefore, it is urgent to solve the problem of apoptosis in ALI/ARDS. Apoptosis of lung endothelial and epithelial cells might initiate or contribute to the progression of many lung diseases [57]. The balance of survival and death of lung endothelial cells is essential for angiogenesis, degeneration, and barrier function. Lung endothelial cells can be damaged by hypoxia or LPS stimulation. However, VEGF is abundant in endothelial cells and can promote endothelial cell survival and maintain normal alveolar structure. [1]. Our previous study also demonstrated that DM could promote the expression of VEGF in lung tissue, thereby protecting lung endothelial cells from apoptosis. The Bcl-2 protein family is an essential regulator of apoptotic cells [58, 59]. Bax is a crucial executor of mitochondrial regulation of cell death, and its dysregulation guides abnormal cell death [60]. PI3K/Akt plays a vital role in regulating anti-inflammatory cytokines, immune regulation, extracellular matrix degradation, proliferation, and apoptosis [61]. Studies have confirmed that the alleviation of lung injury is related to activation of the PI3K/Akt pathway, downregulation of proinflammatory cytokines, and regulation of apoptosis-related proteins (downregulation of caspase-3, caspase-9 and Bax and upregulation of Bcl-2) [62]. In our study, DM preconditioning also downregulated Bax, upregulated Bcl-2 and activated the PI3K/Akt signaling pathway. Therefore, it was revealed that DM could significantly alleviate lung injury by activating PI3K/Akt, promoting proliferation and regulating apoptosis. These findings lay the foundation for further in-depth exploration of the pathological mechanisms of and interventional strategies for lung injury.

## Conclusion

In this study, we first investigated the possible mechanism of DM in treating ALI through a comprehensive strategy combining metabolomics, network pharmacology and molecular biology experiments. The integrated analysis of metabolomics and network pharmacology predicted that DM could treat ALI mainly by improving the inflammatory response and regulating cell apoptosis. The accuracy of the strategy and predictive results were preliminarily validated by biological experiments. The results confirmed that integrated metabolomics and network pharmacology analysis to predict the potential mechanism of DM treatment of ALI is accurate and feasible. This comprehensive strategy provides a more precise direction for exploring the treatment of ALI with natural medicines.

## Supplementary Information

**Additional file 1: Fig. S1.** The stem of *Nauclea officinalis* Pierre ex Pitard (Danmu in Chinese, DM). **Fig. S2.** HPLC analysis of DM.

**Additional file 2: Fig. S3.** The representative total ion chromatograms (TICs) of QC samples on positive and negative ion mode in plasma and lung tissue. PCA score plots and RSD% distribution of QC samples for metabolomic validation. **Fig. S4.** The representative total ion chromatograms (TICs) of plasma and lung tissue samples in positive and negative ion mode. **Fig. S5.** PLS-DA analysis of plasma and lung tissue in mice. **Fig. S6.** S-plot analysis of plasma and lung tissue samples in positive and negative ion mode. **Table S1.** Parameters of PLS-DA model and OPLS-DA model for prediction. **Table S2.** Enrichment analysis of potential metabolic pathways in mouse plasma and lung tissue.

**Additional file 3: Table S3. **Summary of chemical constituents in DM. **Table S4.** Active components of DM in the treatment of ALI.

**Additional file 4: **Targets information of DM.

**Additional file 5: Fig. S7.** The core genes for the integrated analysis of metabolomics and network pharmacology.

## Data Availability

The data used and/or investigated during the present study are available from the corresponding author upon reasonable request.

## Abbreviations

ALI: Acute lung injury
DM: *Nauclea officinalis* (Danmu in Chinese, DM)
LPS: Lipopolysaccharide
BALF: Bronchoalveolar lavage fluid
VEGF: Vascular endothelial growth factor
PCA: Principal component analysis
PLS-DA: Partial least squares discriminant analysis
OPLS-DA: Orthogonal projections to latent structures- discriminant analysis
LA: Linoleic acid
AA: Arachidonic acid
PGs: Prostaglandins
LTs: Leukotrienes
COX: Cyclooxygenase
PEs: Phosphatidylethanolamines
PCs: Phosphatidylcholines
PIs: Phosphatidylinositols
PAs: Phosphatidic acids
LPCs: Lyso-phosphatidylcholines
LPEs: Lyso-phosphatidylethanolamines
