# Supplementary material for: Integrated metabolomics, network pharmacology and biological verification to reveal the mechanisms of Nauclea officinalis treatment of LPS-induced acute lung injury
Source: Chin Med. 2022 Nov 24;17:131. doi: 10.1186/s13020-022-00685-6 (PMC9700915; doi:10.1186/s13020-022-00685-6)
Supplement: Supplementary file 1 — Additional file 1: Fig. S1. The stem of Nauclea officinalis Pierre ex Pitard (Danmu in Chinese, DM). Fig. S2. HPLC analysis of DM. [file 13020_2022_685_MOESM1_ESM.docx]

**Materials and Methods**


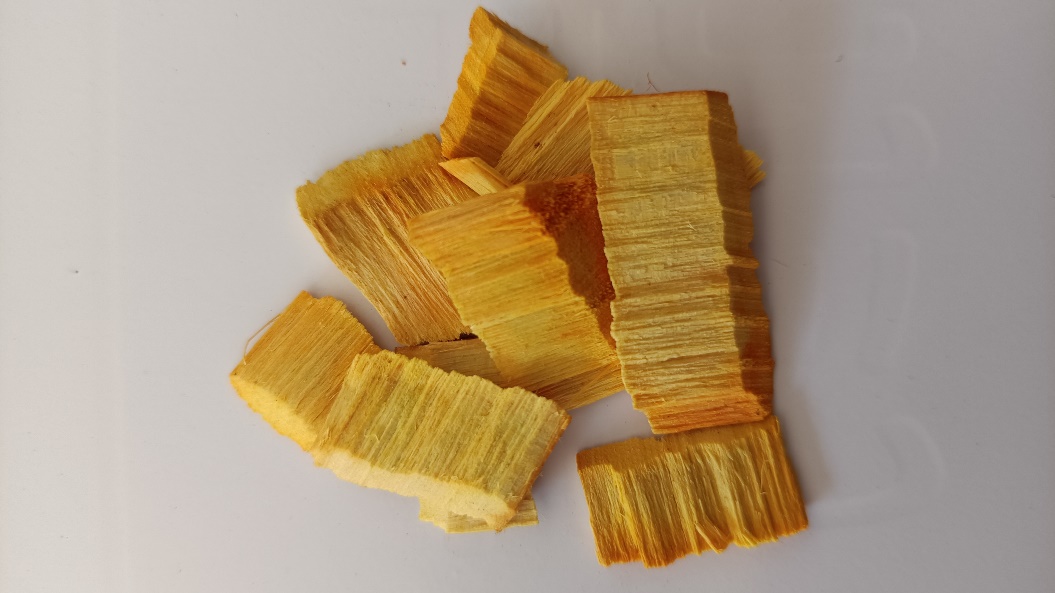


**Additional Figure S1** The stem of *Nauclea officinalis* Pierre *ex* Pitard (Danmu in Chinese, DM)

***HPLC Analysis of DM***

The water extract of *Nauclea officinalis* Pierre *ex* Pitard (Danmu in Chinese, DM) was analyzed by a Waters e2695 liquid chromatography system and the chromatographic column was Waters XDSC18 (150mm×4.6mm, 5μm). the mobile phase was composed of acetonitrile (A) and 0.1% Phosphoric acid (B). The gradient elution conditions were as follows: 0-20 min, 5% A; 20-60 min, 5-28% A; 60-100 min, 28-70% A; 100-110 min, 70% A. The column temperature was set at 40 °C, the flow rate was 1 ml/min, and the wavelength of 280 nm. Moreover, the contents of Chlorogenic acid, Strictosamide and Vincosamide in DM were Determined. Before injection, all samples were filtered by 0.45μm membrane.

**Results**

***Analysis of chemical constituents of DM extract***

In this study, HPLC technique was used to rapidly analyze and identify the chemical constituents of DM. The HPLC chromatogram of the DM extract is shown in Supplement Figure 2A. As show in Supplement Figure 2B, the contents of Chlorogenic acid, Strictosamide and Vincosamide in DM were 5.72%, 12.91% and 0.33%, respectively.


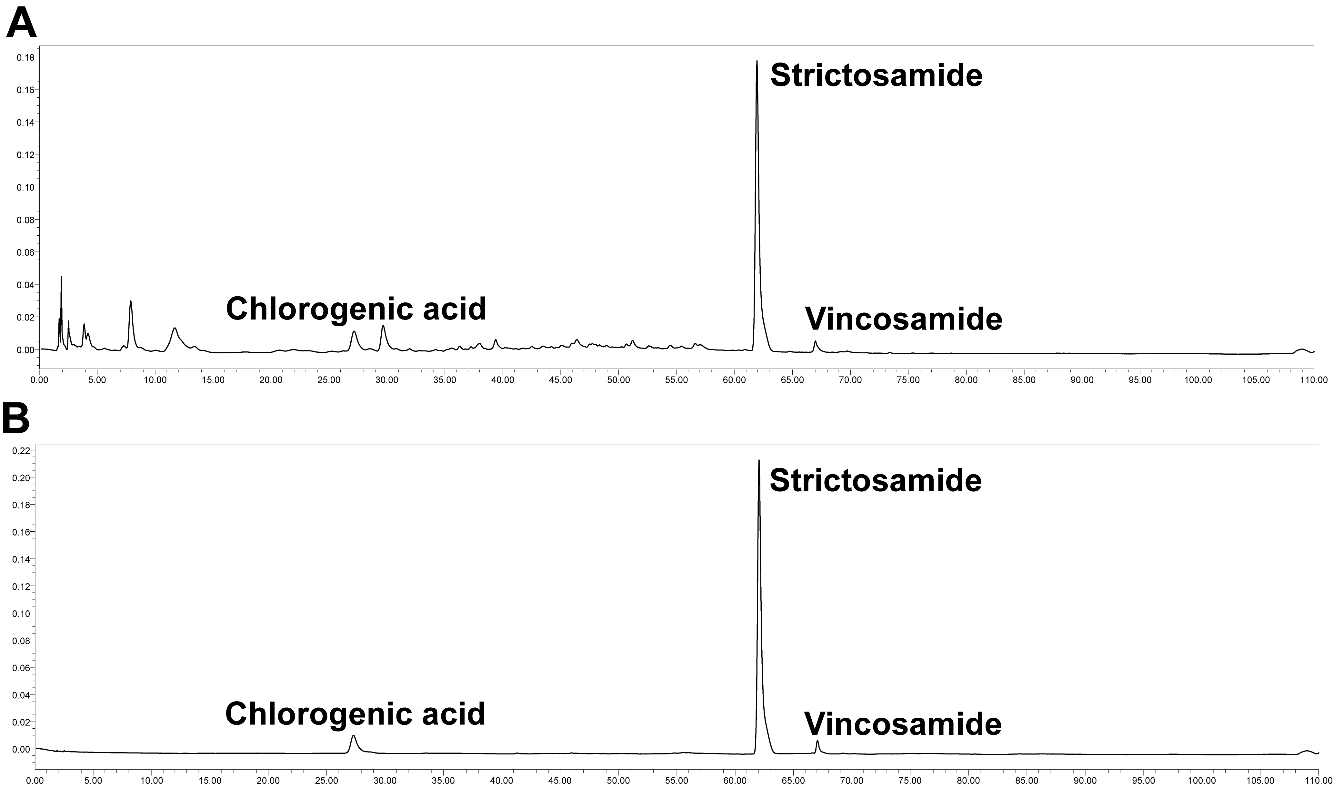


**Additional Figure S2** HPLC analysis of DM. (A) HPLC pattern of DM extract, (B) Determination of Chlorogenic acid, Strictosamide and Vincosamide at 280 nm.
