# Supplementary material for: Integrated metabolomics, network pharmacology and biological verification to reveal the mechanisms of Nauclea officinalis treatment of LPS-induced acute lung injury
Source: Chin Med. 2022 Nov 24;17:131. doi: 10.1186/s13020-022-00685-6 (PMC9700915; doi:10.1186/s13020-022-00685-6)
Supplement: Supplementary file 2 — Additional file 2: Fig. S3. The representative total ion chromatograms (TICs) of QC samples on positive and negative ion mode in plasma and lung tissue. PCA score plots and RSD% distribution of QC samples for metabolomic validation. Fig. S4. The representative total ion chromatograms (TICs) of plasma and lung tissue samples in positive and negative ion mode. Fig. S5. PLS-DA analysis of plasma and lung tissue in mice. Fig. S6. S-plot analysis of plasma and lung tissue samples in positive and negative ion mode. Table S1. Parameters of PLS-DA model and OPLS-DA model for prediction. Table S2. Enrichment analysis of potential metabolic pathways in mouse plasma and lung tissue. [file 13020_2022_685_MOESM2_ESM.docx]

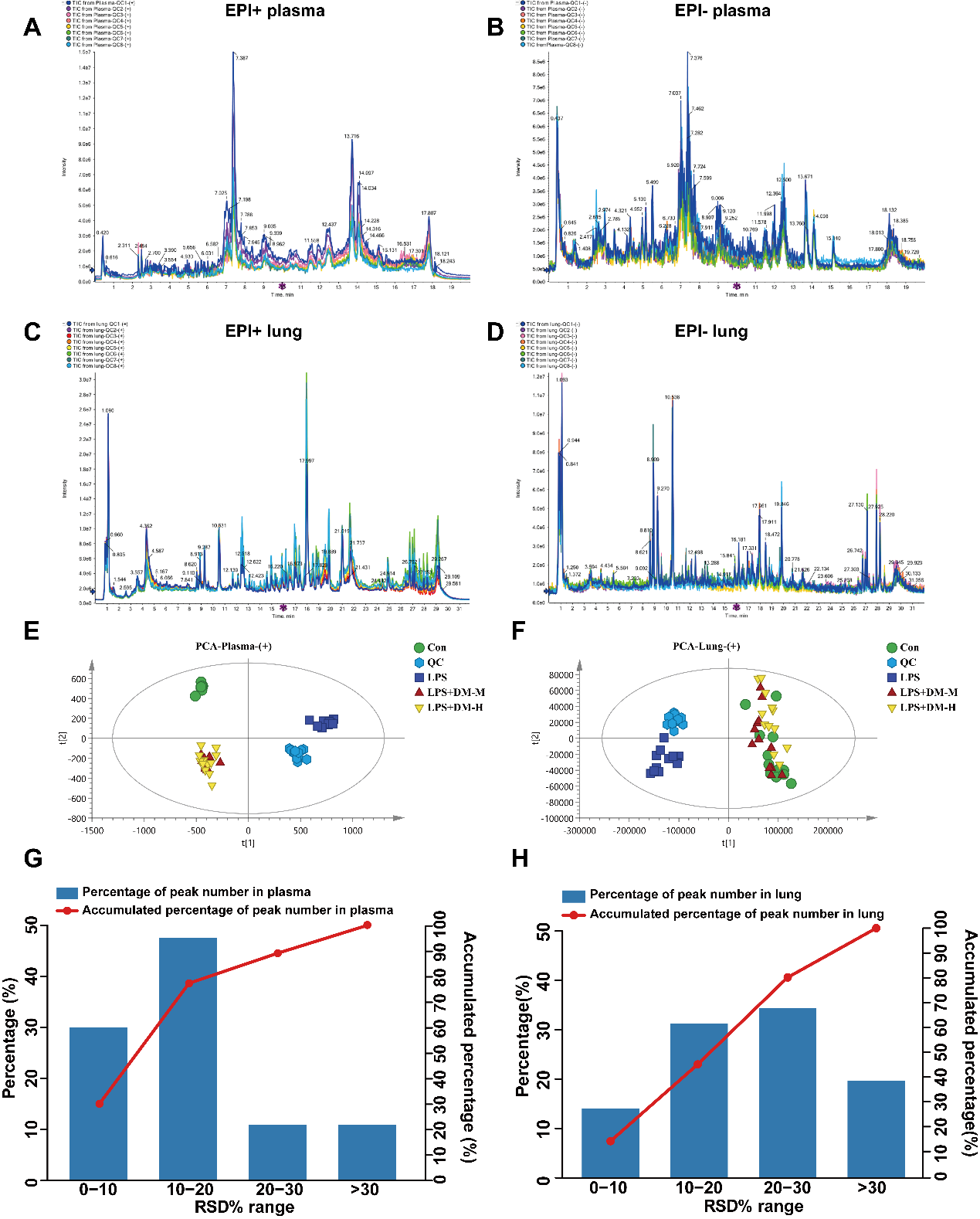


**Additional Figure S3** The representative total ion chromatograms (TICs) of QC samples on positive and negative ion mode in plasma (A, B) and lung tissue (C, D). PCA score plots (E and F) and RSD% distribution (G and H) of QC samples for metabolomic validation.


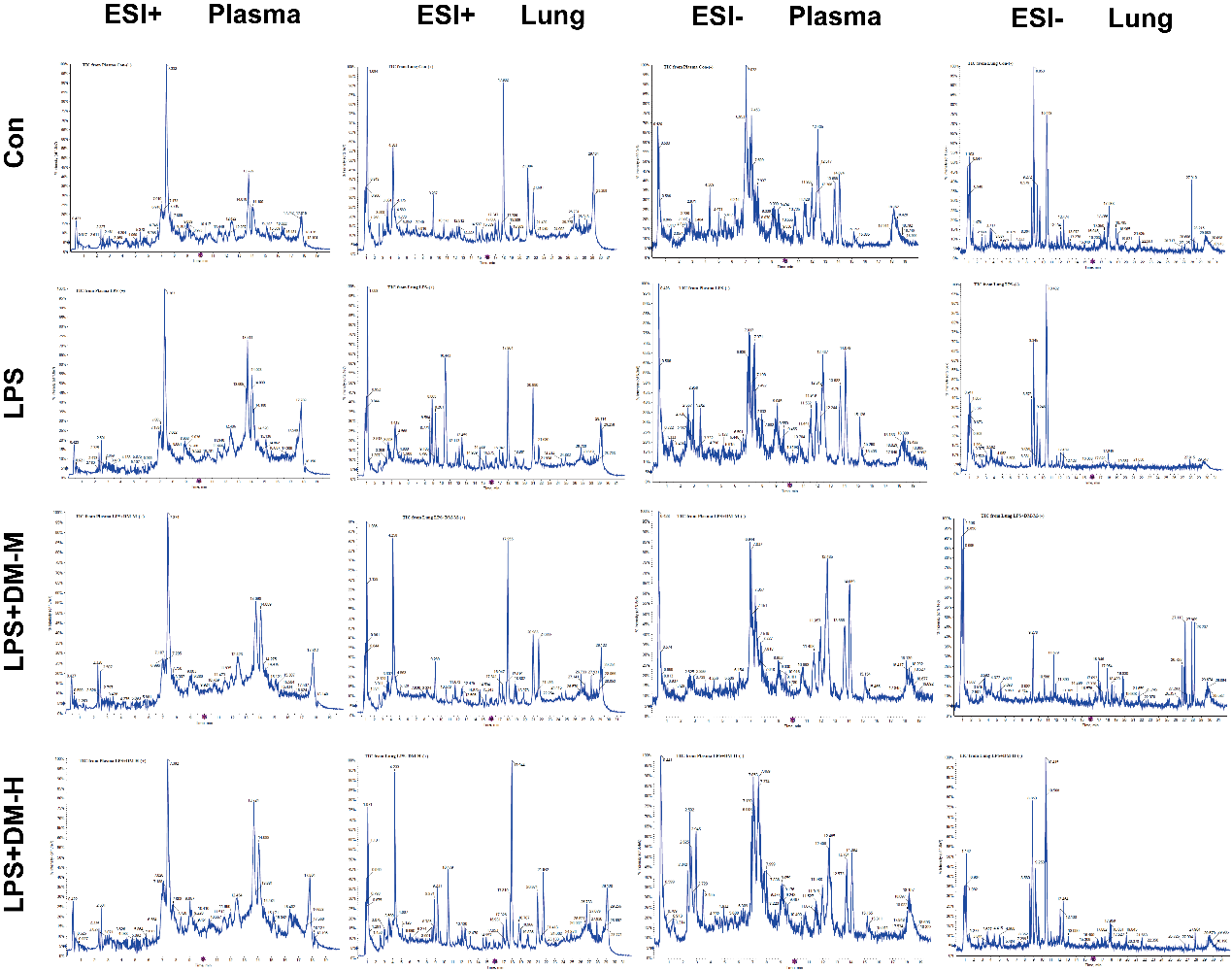


**Additional Figure S4** The representative total ion chromatograms (TICs) of plasma and lung tissue samples in positive and negative ion mode.


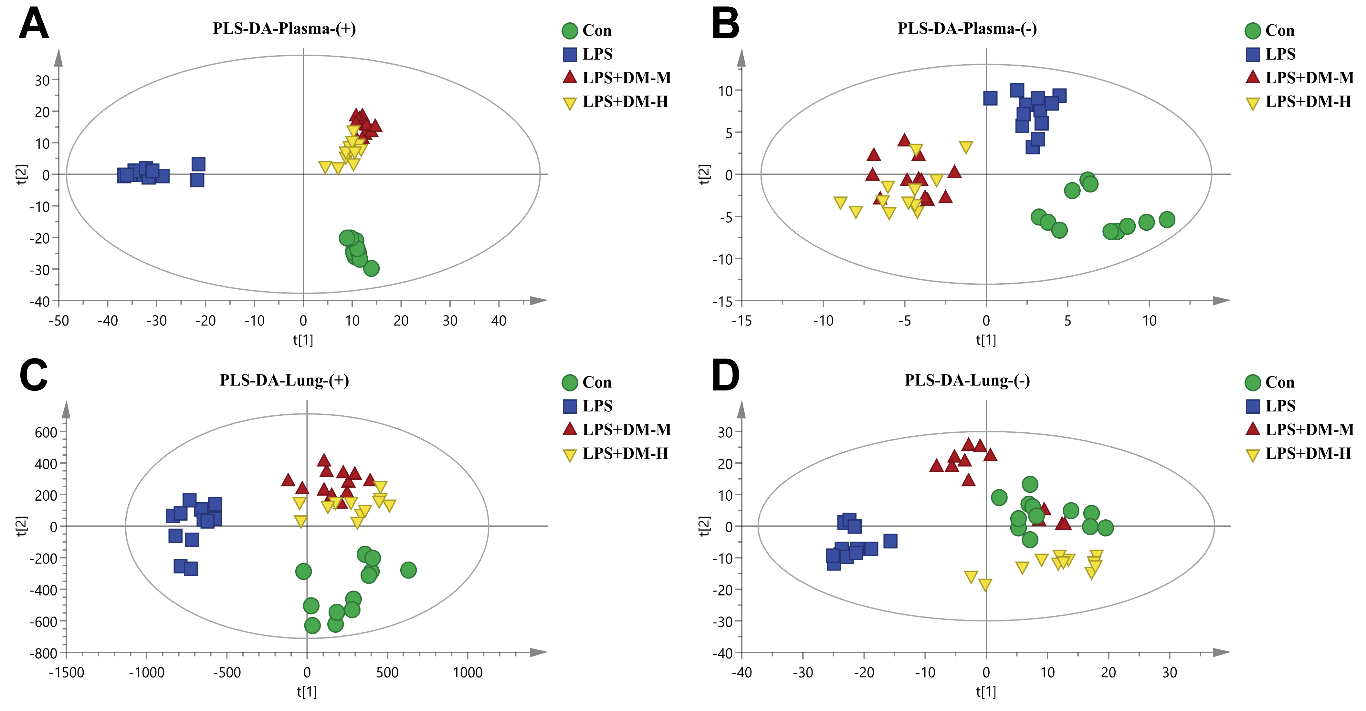


**Additional Figure S5** PLS-DA analysis of plasma and lung tissue in mice. (A) Plasma positive ion mode PLS-DA diagram; (B) Plasma negative ion mode PL-SDA diagram; (C) Lung tissue positive ion mode PLS-DA diagram; (D) Lung tissue negative ion mode PLS-DA diagram.


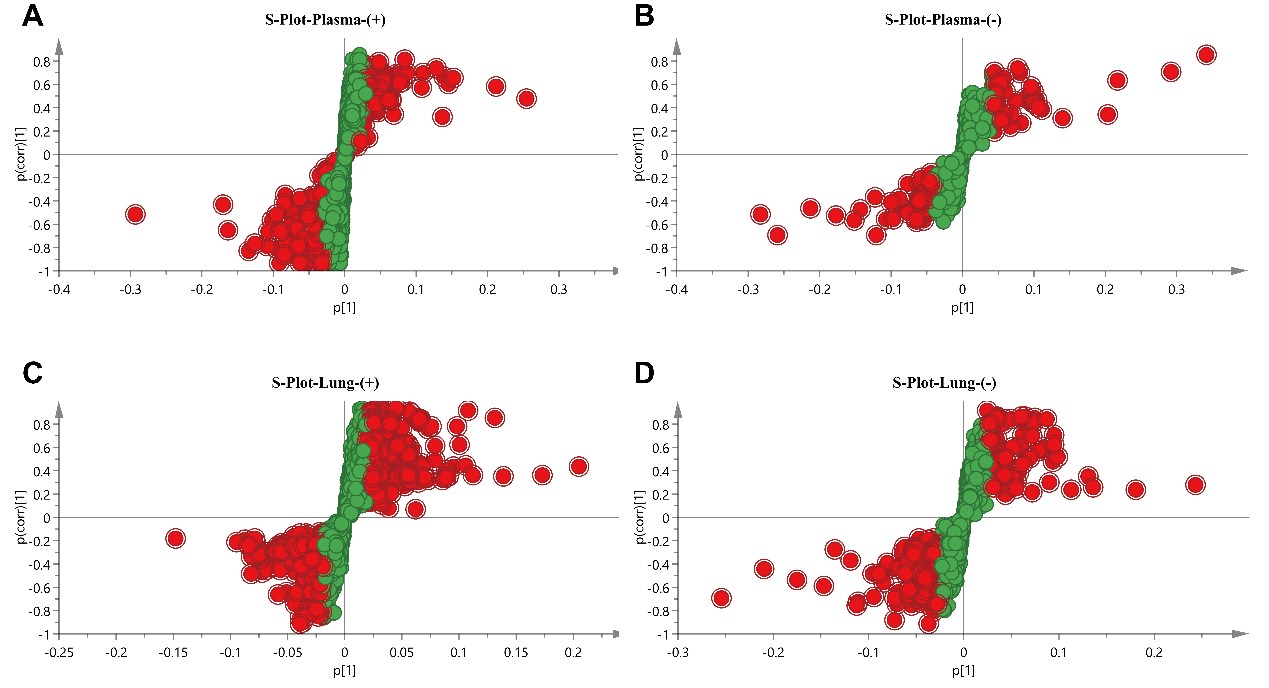


**Additional Figure S6** S-plot analysis of plasma (A and B) and lung tissue (C and D) samples in positive and negative ion mode.

**Additional Table S1** Parameters of PLS-DA model and OPLS-DA model for prediction.

| Model | Positive | | | Negative | | | Part |
| --- | --- | --- | --- | --- | --- | --- | --- |
|  | R^2^ X | R^2^Y | Q^2^ | R^2^ X | R^2^Y | Q^2^ |  |
| PLS-DA | 0.554 | 0.987 | 0.921 | 0.595 | 0.978 | 0.608 | Plasma |
| PLS-DA | 0.625 | 0.959 | 0.792 | 0.721 | 0.943 | 0.583 | Lung |
| OPLS-DA | 0.572 | 0.992 | 0.956 | 0.795 | 0.995 | 0.675 | Plasma |
| OPLS-DA | 0.695 | 0.998 | 0.811 | 0.951 | 0.963 | 0.692 | Lung |

**Additional Table S2** Enrichment analysis of potential metabolic pathways in mouse plasma and lung tissue.

| NO. | Pathway name | *P* | -log (p) | Impact | Part |
| --- | --- | --- | --- | --- | --- |
| 1 | Linoleic acid metabolism | 0.00091 | 3.04070 | 1.00000 | Plasma |
| 2 | Arachidonic acid metabolism | 0.04800 | 1.31880 | 0.33292 | Plasma |
| 3 | Glycerophospholipid metabolism | 0.04800 | 1.31880 | 0.19895 | Plasma |
| 4 | Sphingolipid metabolism | 0.01744 | 1.75850 | 0.15822 | Plasma |
| 5 | Ether lipid metabolism | 0.18248 | 0.73878 | 0.14458 | Plasma |
| 6 | Primary bile acid biosynthesis | 0.37345 | 0.42777 | 0.05517 | Plasma |
| 7 | Pantothenate and CoA biosynthesis | 0.17415 | 0.75907 | 0.00714 | Plasma |
| 8 | Glycosylphosphatidylinositol (GPI)-anchor biosynthesis | 0.13130 | 0.88175 | 0.00399 | Plasma |
| 9 | Biosynthesis of unsaturated fatty acids | 0.00678 | 2.1688 | 0 | Plasma |
| 10 | Alpha-Linolenic acid metabolism | 0.13772 | 0.861 | 0 | Plasma |
| 11 | Histidine metabolism | 0.16686 | 0.77765 | 0 | Plasma |
| 12 | Pyrimidine metabolism | 0.36139 | 0.44203 | 0 | Plasma |
| 13 | Ubiquinone and other terpenoid-quinone biosynthesis | 0.10283 | 0.98789 | 1.00000 | Lung |
| 14 | Purine metabolism | 0.18485 | 0.73319 | 0.18623 | Lung |
| 15 | Arginine and proline metabolism | 0.00082 | 3.0864 | 0.12393 | Lung |
| 16 | Tyrosine metabolism | 0.40074 | 0.39714 | 0.08276 | Lung |
| 17 | Vitamin B6 metabolism | 0.10283 | 0.98789 | 0.07843 | Lung |
| 18 | beta-Alanine metabolism | 0.024778 | 1.6059 | 0.05597 | Lung |
| 19 | Glutathione metabolism | 0.00390 | 2.409 | 0.03417 | Lung |
| 20 | Cysteine and methionine metabolism | 0.33042 | 0.48094 | 0.02659 | Lung |
| 21 | Arachidonic acid metabolism | 0.35468 | 0.45016 | 0.0212 | Lung |
| 22 | Glycerophospholipid metabolism | 0.35468 | 0.45016 | 0.01736 | Lung |
| 23 | Phenylalanine, tyrosine and tryptophan biosynthesis | 0.047004 | 1.3279 | 0 | Lung |
| 24 | Retinol metabolism | 0.17581 | 0.75495 | 0 | Lung |
| 25 | Glycine, serine and threonine metabolism | 0.3386 | 0.47031 | 0 | Lung |
| 26 | Primary bile acid biosynthesis | 0.4297 | 0.36683 | 0 | Lung |
| 27 | Aminoacyl-tRNA biosynthesis | 0.44368 | 0.35293 | 0 | Lung |
