# Supplementary material for: Integrated metabolomics, network pharmacology and biological verification to reveal the mechanisms of Nauclea officinalis treatment of LPS-induced acute lung injury
Source: Chin Med. 2022 Nov 24;17:131. doi: 10.1186/s13020-022-00685-6 (PMC9700915; doi:10.1186/s13020-022-00685-6)
Supplement: Supplementary file 3 — Additional file 3: Table S3. Summary of chemical constituents in DM. Table S4. Active components of DM in the treatment of ALI. [file 13020_2022_685_MOESM3_ESM.docx]

**Additional Table S3** Summary of chemical constituents in DM.

| NO. | Compounds | Compound CID | Compound CAS | Species |
| --- | --- | --- | --- | --- |
| 1 | Naucleosides A | NA | NA | Alkaloids |
| 2 | Naucleonine | 5320035 | NA | Alkaloids |
| 3 | Naucleofficine I | NA | NA | Alkaloids |
| 4 | Naucleofficine H | NA | NA | Alkaloids |
| 5 | Naucleofficine F | NA | NA | Alkaloids |
| 6 | Naucleofficine E | NA | NA | Alkaloids |
| 7 | Naucleofficine D | 101855146 | NA | Alkaloids |
| 8 | Naucleofficine C | 101855145 | NA | Alkaloids |
| 9 | Nectandrin A | 13939326 | 477-60-1 | Alkaloids |
| 10 | Naucleofficine B | 24862702 | NA | Alkaloids |
| 11 | β-Naucleonidine | 10969739 | 55221-44-8 | Alkaloids |
| 12 | α-naucleonidine | 10759308 | NA | Alkaloids |
| 13 | Vinmajine I | 122206354 | NA | Alkaloids |
| 14 | Vincoside lactam | 44567645 | 23141-27-7 | Alkaloids |
| 15 | Vincosamide | 10163855 | 23141-27-7 | Alkaloids |
| 16 | Vanillic acid | 8468 | 121-34-6 | Alkaloids |
| 17 | Tetrahydrodesoxycordifoline | NA | NA | Alkaloids |
| 18 | Strictosamide | 10345799 | 23141-25-5 | Alkaloids |
| 19 | Quinic acid | 6508 | 77-95-2 | Alkaloids |
| 20 | Parvine | 320217 | NA | Alkaloids |
| 21 | Paratunamide E | NA | NA | Alkaloids |
| 22 | Paratunamide D | 73240856 | NA | Alkaloids |
| 23 | Paratunamide C | NA | 914778-25-9 | Alkaloids |
| 24 | Paratunamide A | NA | 914778-23-7 | Alkaloids |
| 25 | Naulafine | 14313083 | 70503-66-1 | Alkaloids |
| 26 | Naufoline | 101223026 | NA | Alkaloids |
| 27 | Naucxlequiniine | NA | NA | Alkaloids |
| 28 | Naucxlefiline | NA | NA | Alkaloids |
| 29 | Naucline | 102193811 | NA | Alkaloids |
| 30 | Nauclexine | 608079 | NA | Alkaloids |
| 31 | Naucletine | 5320037 | 54698-29-2 | Alkaloids |
| 32 | Nauclequiniine | 5320036 | NA | Alkaloids |
| 33 | Naucleoxoside A | NA | NA | Alkaloids |
| 34 | Naucleofficine A | 24850489 | NA | Alkaloids |
| 35 | Naucleidinal | 10969739 | 77513-45-2 | Alkaloids |
| 36 | Nauclefoline | 54714261 | 96400-51-0 | Alkaloids |
| 37 | Nauclefine | 320217 | 57103-51-2 | Alkaloids |
| 38 | Nauclefiline | 6438926 | 102358-19-0 | Alkaloids |
| 39 | Nauclefidine | 5320028 | 96400-52-1 | Alkaloids |
| 40 | Naucleficine | 5320030 | 96400-54-3 | Alkaloids |
| 41 | Naucledine | 627757 | 26238-84-6 | Alkaloids |
| 42 | Nauclederine | 624378 | 8940-72-6 | Alkaloids |
| 43 | Nauclecosidine | 129524 | 121880-13-5 | Alkaloids |
| 44 | Nauclecoside | 195350 | 21880-11-3 | Alkaloids |
| 45 | Nauclechine | 632456 | 38940-73-7 | Alkaloids |
| 46 | Naucleamide G | 132941537 | NA | Alkaloids |
| 47 | Naucleamide F | 102283795 | NA | Alkaloids |
| 48 | Naucleamide E | 12047482 | NA | Alkaloids |
| 49 | Naucleamide D | 11035072 | NA | Alkaloids |
| 50 | Naucleamide C | 12047480 | NA | Alkaloids |
| 51 | Naucleamide A-10-O-β-D-glucopyranoside | NA | NA | Alkaloids |
| 52 | Naucleamide A | 12047478 | NA | Alkaloids |
| 53 | Nauclealotide C | NA | NA | Alkaloids |
| 54 | Nauclealotide A | NA | NA | Alkaloids |
| 55 | Naucleactonin D | 71545910 | NA | Alkaloids |
| 56 | Naucleactonin A | 101579601 | 946135-01-9 | Alkaloids |
| 57 | Nauckedine | NA | NA | Alkaloids |
| 58 | Loganic acid | 89640 | 22255-40-9 | Alkaloids |
| 59 | Latifoliamide D | NA | NA | Alkaloids |
| 60 | Latifoliamide B | NA | NA | Alkaloids |
| 61 | Latifoliamide A | NA | NA | Alkaloids |
| 62 | Harmane | 5281404 | 486-84-0 | Alkaloids |
| 63 | Epimethoxynaucleaorine | NA | NA | Alkaloids |
| 64 | Desoxycordifolinic acid | 5316605 | 88839-94-5 | Alkaloids |
| 65 | Desoxycordifoline | 21577866 | 21451-50-3 | Alkaloids |
| 66 | Decarbomethoxynauclechine | NA | 59785-76-1 | Alkaloids |
| 67 | β-Carboline-1-carboxylic acid | 98375 | 26052-96-0 | Alkaloids |
| 68 | Cadambine | 398038 | 54422-49-0 | Alkaloids |
| 69 | Barbinervic acid | 194183 | 64199-78-6 | Alkaloids |
| 70 | Angustine | 441983 | 40041-96-1 | Alkaloids |
| 71 | Angustidine | 3084770 | 40217-50-3 | Alkaloids |
| 72 | 6-O-acetylstritosamide | 5316111 | NA | Alkaloids |
| 73 | 5-β-carboxystrictosidine | 10483216 | NA | Alkaloids |
| 74 | 3α-dihydrocadambine | 162138 | 54483-84-0 | Alkaloids |
| 75 | 3α-5α-tetrahydrodeoxycordifoline lactam | NA | NA | Alkaloids |
| 76 | 3α,3β-Carboxyvincoside lactam | NA | NA | Alkaloids |
| 77 | 3,14-dihydroangustoline | 101629128 | NA | Alkaloids |
| 78 | 3,14-dihydroangustine | 176611 | NA | Alkaloids |
| 79 | 3,14,18,19-tetrahydroangustine | 101629127 | NA | Alkaloids |
| 80 | 1-acetyl-β-carboline | NA | 50892-83-6 | Alkaloids |
| 81 | 19-O-Methylangustoline | 5319508 | NA | Alkaloids |
| 82 | 19-O-Ethylangustoline | 15336905 | NA | Alkaloids |
| 83 | 18, 19- dihydroangustine | 5316691 | NA | Alkaloids |
| 84 | 17-epinaucleidinal | 50987801 | 77513-46-3 | Alkaloids |
| 85 | 10-hydroxystrictosamide | 70675008 | NA | Alkaloids |
| 86 | 10-hydroxyangustine | 5318147 | NA | Alkaloids |
| 87 | 1,2,3,4-tetrahydronorharman-1-one | 87371 | 17952-82-8 | Alkaloids |
| 88 | Naucleamide B | 12047479 | NA | Alkaloids |
| 89 | Naucleamide A | 12047478 | NA | Alkaloids |
| 90 | Naucleactonin B | 101579602 | NA | Alkaloids |
| 91 | Angustoline | 3084765 | 40041-95-0 | Alkaloids |
| 92 | Pumiloside | 10346314 | 126722-26-7 | Alkaloids |
| 93 | 3-epi-Pumiloside | NA | 126624-21-3 | Alkaloids |
| 94 | 1,2,3,4-tetrahydro-β-carboline | 46783042 | 16502-01-5 | Alkaloids |
| 95 | 3-R-3,4-dihydroangustoline | NA | NA | Alkaloids |
| 96 | 3-S-3,4-dihydroangustoline | NA | NA | Alkaloids |
| 97 | Ethyl 3, 4-dihydroxycinnamate | 5468519 | 66648-50-8 | Phenolic acids |
| 98 | (+) – medioresinol | 181681 | 40957-99-1 | Phenolic acids |
| 99 | 2, 3-Dihydroxybenzoic acid | 19 | 303-38-8 | Phenolic acids |
| 100 | 2,5-Dimethoxybenzoic acid | 76027 | 2785-98-0 | Phenolic acids |
| 101 | 3, 4, 5-trimethoxybenzoic acid | 8357 | 118-41-2 | Phenolic acids |
| 102 | 3, 4, 5-trimethoxyphenyl-β-D-glucopyranoside | NA | 109206-94-2 | Phenolic acids |
| 103 | 3,4,5 -trimethylphenol | 10696 | 527-54-8 | Phenolic acids |
| 104 | 3, 4-Dimethoxycinnamic acid | 717531 | 2316-26-9 | Phenolic acids |
| 105 | Methyl 3,4-Dihydroxybenzoate | 287064 | 2150-43-8 | Phenolic acids |
| 106 | 3,4-dihydroxycinnamic acid | 689043 | 331-39-5 | Phenolic acids |
| 107 | 3,4-dimethoxycinnamic acid | 717531 | 2316-26-9 | Phenolic acids |
| 108 | 3,4-dimethoxyphenol-β-D-apiofuranosyl (1-6)-β-D-glucopyranoside | | NA | Phenolic acids |
| 109 | 3,4,5-trihydroxybenzoic acid | 370 | 149-91-7 | Phenolic acids |
| 110 | 3-O-Acetylerythrodiol | 118796402 | NA | Phenolic acids |
| 111 | 4-hydroxy-3,5-dimethoxybenzaldehyde | 8655 | 134-96-3 | Phenolic acids |
| 112 | Bis-(2-ethylhexyl) phthalate | 8343 | 117-81-7 | Phenolic acids |
| 113 | Blumenol A | 5280462 | 23526-45-6 | Phenolic acids |
| 114 | Caffeic acid methyl ester | 689075 | 3843-74-1 | Phenolic acids |
| 115 | Beta-sitosterol palmitate | 13747834 | 84414-74-4 | Phenolic acids |
| 116 | Chlorogenic acid | 1794427 | 327-97-9 | Phenolic acids |
| 117 | Cinnamic acid | 444539 | 621-82-9 | Phenolic acids |
| 118 | Cryptochlorogenic acid | 9798666 | 905-99-7 | Phenolic acids |
| 119 | Di-(2-ethylhexyl) phthalate | 8343 | 117-81-7 | Phenolic acids |
| 120 | Ethyl gallate | 13250 | 831-61-8 | Phenolic acids |
| 121 | Ethyl caffeate | 5317238 | 102-37-4 | Phenolic acids |
| 122 | Gallic acid | 370 | 149-91-7 | Phenolic acids |
| 123 | Glycosmisic acid | 38356815 | 443908-19-8 | Phenolic acids |
| 124 | Kelampayoside A | 10552637 | 87562-76-3 | Phenolic acids |
| 125 | Kaempferol-3-O-β-D-glucopyranoside | NA | 480-10-4 | Phenolic acids |
| 126 | Khaephuoside A | 15747353 | NA | Phenolic acids |
| 127 | Methyl isoferulate | 6439893 | 16980-82-8 | Phenolic acids |
| 128 | Neochlorogenic acid | 5280633 | 906-33-2 | Phenolic acids |
| 129 | P-hydroxybenzoic acid | 135 | 99-96-7 | Phenolic acids |
| 130 | p-methoxy cinnamic acid | 13245 | 830-09-1 | Phenolic acids |
| 131 | Protocatechuic acid | 72 | 99-50-3 | Phenolic acids |
| 132 | Resveratrol | 445154 | 501-36-0 | Phenolic acids |
| 133 | Vanillin | 1183 | 121-33-5 | Phenolic acids |
| 134 | 9,19-cyclolanost-24-en-3-one | 51041311 | 511-63-7 | Terpenoids |
| 135 | Diderroside | 23760099 | 86989-19-7 | Terpenoids |
| 136 | Dihydroactinidiolide | 27209 | 17092-92-1 | Terpenoids |
| 137 | Hederagenin | 73299 | 465-99-6 | Terpenoids |
| 138 | Loganin | 87691 | 18524-94-2 | Terpenoids |
| 139 | Loliolide | 100332 | 5989-02-6 | Terpenoids |
| 140 | Marounoside | 101927055 | NA | Terpenoids |
| 141 | Naucledal | 101306866 | 38965-50-3 | Terpenoids |
| 142 | Naucleol | 5367539 | 38965-49-0 | Terpenoids |
| 143 | Oleanlic acid | NA | 508-02-1 | Terpenoids |
| 144 | Quinovic acid | 120678 | 465-74-7 | Terpenoids |
| 145 | Secologanoside | 14136854 | 59472-23-0 | Terpenoids |
| 146 | Secoxyloganin | 162868 | 58822-47-2 | Terpenoids |
| 147 | Sweroside | 161036 | 14215-86-2 | Terpenoids |
| 148 | Ursolic aldehyde | 14423519 | 19132-81-1 | Terpenoids |
| 149 | Glycosmisic acid | 38356815 | 443908-19-8 | Flavonoids |
| 150 | Hesperidin | 10621 | 520-26-3 | Flavonoids |
| 151 | Kaempferol-3-O-rutinoside | 5318767 | 17650-84-9 | Flavonoids |
| 152 | Rutin | 5280805 | 153-18-4 | Flavonoids |
| 153 | Rutinum | 5280805 | 153-18-4 | Flavonoids |
| 154 | 24-en-cycloartenone | NA | NA | Others |
| 155 | Vincoline | 78358513 | 11034-66-5 | Others |
| 156 | Carotin | 5280489 | 7235-40-7 | Others |
| 157 | Daucosterol | 742590 | 474-58-8 | Others |
| 158 | Loganin | 87691 | 18524-94-2 | Others |
| 159 | Sitosterone | 9801811 | 51529-11-4 | Others |
| 160 | Stigmast-4-en-3-one | 5484202 | 1058-61-3 | Others |
| 161 | Stigmastane-3,6-dione | 13992093 | 22149-69-5 | Others |
| 162 | Sweroside | 161036 | 14215-86-2 | Others |
| 163 | Vanillin | 1183 | 121-33-5 | Others |
| 164 | Veratric acid | 7121 | 93-07-2 | Others |
| 165 | β-Sitosterol | 222284 | 83-46-5 | Others |
| 166 | β-sitosterol palmitate | 9852570 | NA | Others |

**Additional Table S4** Active components of DM in the treatment of ALI.

| NO. | Molecule name | Degree | Betweenness | Closeness |
| --- | --- | --- | --- | --- |
| D24 | Barbinervic acid | 82 | 1.50E-04 | 1 |
| D13 | Ethyl caffeate | 76 | 2.03E-04 | 1 |
| D4 | hederagenin | 70 | 1.20E-04 | 1 |
| D1 | daucosterol | 66 | 1.17E-04 | 1 |
| D9 | Loganin | 61 | 1.72E-04 | 1 |
| D27 | resveratrol | 60 | 1.42E-04 | 1 |
| D23 | β-sitosterol palmitate | 52 | 1.04E-04 | 1 |
| D21 | vincoside lactam | 49 | 8.71E-05 | 1 |
| D28 | Strictosamide | 49 | 8.71E-05 | 1 |
| D11 | β-sitosterol | 44 | 5.24E-05 | 1 |
| D8 | stigmast-4-en-3-one | 43 | 7.18E-05 | 1 |
| D18 | Veratric acid | 43 | 1.08E-04 | 1 |
| D17 | Harmane | 36 | 1.15E-04 | 1 |
| D15 | Neochlorogenic acid | 33 | 9.17E-05 | 1 |
| D16 | Quinic acid | 33 | 7.47E-05 | 1 |
| D12 | cinnamic acid | 30 | 5.39E-05 | 1 |
| D3 | vanillic acid | 26 | 3.67E-05 | 1 |
| D26 | 9,19-cyclolanost-24-en-3-one | 25 | 2.32E-05 | 1 |
| D19 | hesperidin | 24 | 2.89E-05 | 1 |
| D10 | Chlorogenic acid | 23 | 2.76E-05 | 1 |
| D29 | Vincoline | 20 | 5.54E-05 | 1 |
| D2 | protocatechuic acid | 19 | 3.03E-05 | 1 |
| D6 | 3,4,5-trihydroxybenzoic acid | 19 | 2.68E-05 | 1 |
| D5 | rutin | 18 | 2.57E-05 | 1 |
| D20 | kaempferol-3-O-β-D-glucopyranoside | 18 | 2.54E-05 | 1 |
| D31 | Nauclefiline | 16 | 3.39E-05 | 1 |
| D7 | vanillin | 14 | 2.24E-05 | 1 |
| D22 | (+)-medioresinol | 9 | 2.62E-05 | 1 |
| D30 | (-)-Nectandrin A | 7 | 2.37E-05 | 1 |
| D25 | 3,4,5 -trimethylphenol | 5 | 5.00E-06 | 1 |
| D14 | Secoxyloganin | 3 | 7.31E-06 | 1 |
