# Supplementary material for: Integrated metabolomics, network pharmacology and biological verification to reveal the mechanisms of Nauclea officinalis treatment of LPS-induced acute lung injury
Source: Chin Med. 2022 Nov 24;17:131. doi: 10.1186/s13020-022-00685-6 (PMC9700915; doi:10.1186/s13020-022-00685-6)

**Additional Figure S7** The core genes for the integrated analysis of metabolomics and network pharmacology.


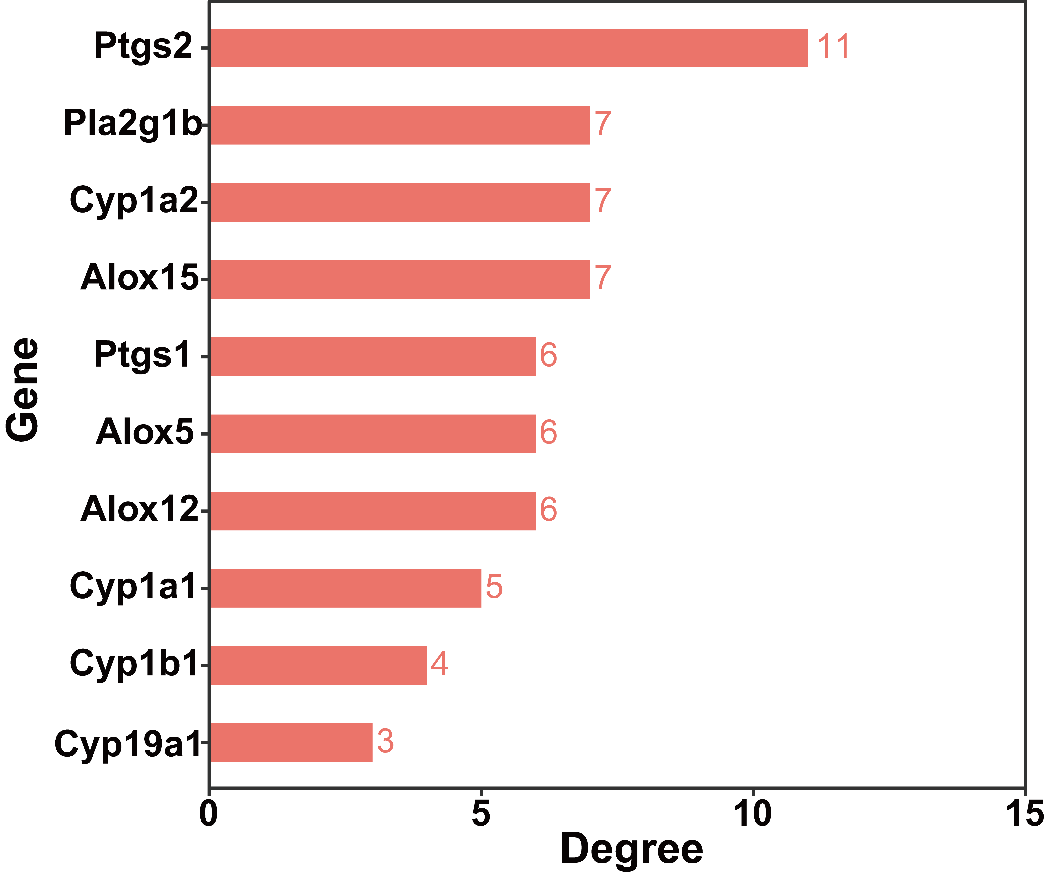

Supplement: Supplementary file 5 — Additional file 5: Fig. S7. The core genes for the integrated analysis of metabolomics and network pharmacology. [file 13020_2022_685_MOESM5_ESM.docx]
